# Supplementary material for: Interpretation of morphogen gradients by a synthetic bistable circuit
Source: Nat Commun. 2020 Nov 2;11:5545. doi: 10.1038/s41467-020-19098-w (PMC7608687; doi:10.1038/s41467-020-19098-w)
Supplement: Supplementary file 1 — Supplementary Information [file 41467_2020_19098_MOESM1_ESM.pdf]

Supplementary Information

Interpretation of morphogen gradients by a synthetic bistable circuit

Paul K. Grant[1], Gregory Szep[1,2,9], Om Patange[3,7,8,9], Jacob Halatek[1], Valerie Coppard[1], Attila Csikász-Nagy[2,4], Jim Haseloff[5], James C. W. Locke[1,3,6], Neil Dalchau[1], and Andrew Phillips[1]

[1]Microsoft Research, 21 Station Road, Cambridge CB1 2FB, UK
[2]Randall Centre for Cell and Molecular Biophysics, King's College London WC2R 2LS, London, UK
[3]Sainsbury Laboratory, University of Cambridge, Cambridge CB2 1LR, UK
[4]Faculty of Information Technology and Bionics, Pázmány Péter Catholic University, 1083 Budapest, Hungary
[5]Department of Plant Sciences, University of Cambridge, Cambridge CB2 3EA, UK
[6]Department of Biochemistry, University of Cambridge, Cambridge CB2 1QW, UK
[7]Present address: Department of Molecular Biology, Massachusetts General Hospital, Boston, MA 02114, USA
[8]Present address: Department of Genetics, Harvard Medical School, Boston, MA 02115, USA
[9]These authors contributed equally: Gregory Szep, Om Patange

Contents

1 Supplementary Figures **2**

2 Supplementary Methods **18**
2.1 Differential Equation Models and Parameter Inference 19
2.2 Bistability Analysis 40
2.3 Boundary Experiments 42
2.4 Models of the Exclusive Receiver Relay Circuits 50

1 Supplementary Figures

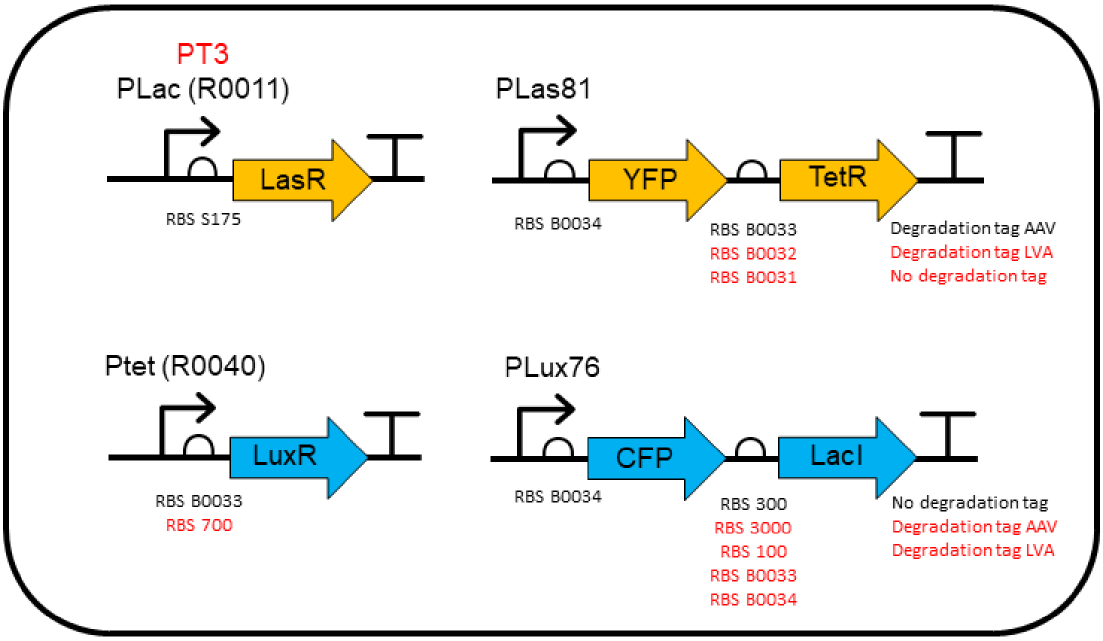

Supplementary Figure 1: Circuit variants. A circuit diagram of the exclusive receiver circuit with genetic parts labelled in black that were included in the final circuit and in red that were evaluated but discarded. Not all combinations of parts were tested.

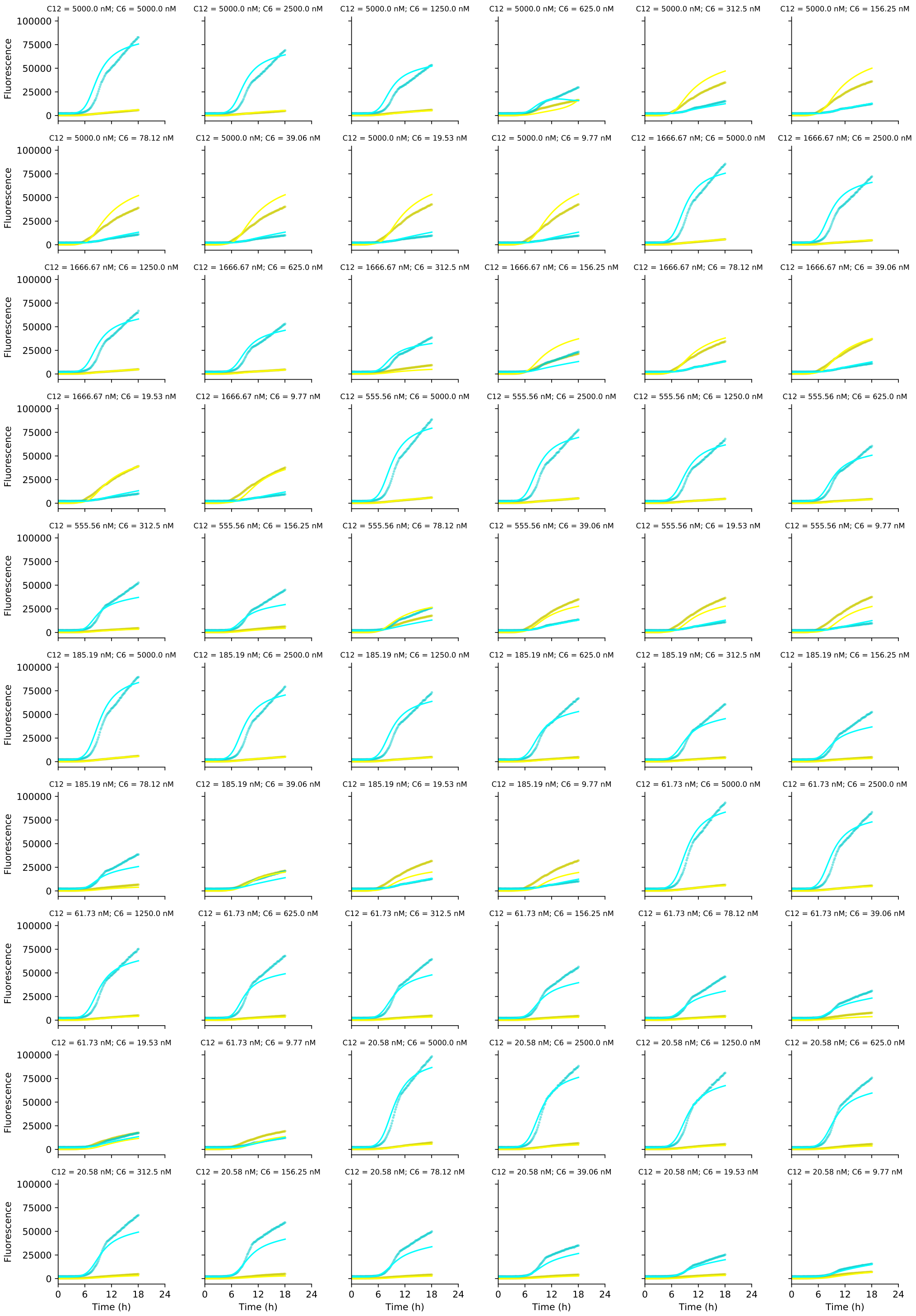
C12 = 5000.0 nM; C6 = 5000.0 nM
C12 = 5000.0 nM; C6 = 2500.0 nM
C12 = 5000.0 nM; C6 = 1250.0 nM
C12 = 5000.0 nM; C6 = 625.0 nM
C12 = 5000.0 nM; C6 = 312.5 nM
C12 = 5000.0 nM; C6 = 156.25 nM
C12 = 5000.0 nM; C6 = 78.12 nM
C12 = 5000.0 nM; C6 = 39.06 nM
C12 = 5000.0 nM; C6 = 19.53 nM
C12 = 5000.0 nM; C6 = 9.77 nM
C12 = 1666.67 nM; C6 = 5000.0 nM
C12 = 1666.67 nM; C6 = 2500.0 nM
C12 = 1666.67 nM; C6 = 1250.0 nM
C12 = 1666.67 nM; C6 = 625.0 nM
C12 = 1666.67 nM; C6 = 312.5 nM
C12 = 1666.67 nM; C6 = 156.25 nM
C12 = 1666.67 nM; C6 = 78.12 nM
C12 = 1666.67 nM; C6 = 39.06 nM
C12 = 1666.67 nM; C6 = 19.53 nM
C12 = 1666.67 nM; C6 = 9.77 nM
C12 = 555.56 nM; C6 = 5000.0 nM
C12 = 555.56 nM; C6 = 2500.0 nM
C12 = 555.56 nM; C6 = 1250.0 nM
C12 = 555.56 nM; C6 = 625.0 nM
C12 = 555.56 nM; C6 = 312.5 nM
C12 = 555.56 nM; C6 = 156.25 nM
C12 = 555.56 nM; C6 = 78.12 nM
C12 = 555.56 nM; C6 = 39.06 nM
C12 = 555.56 nM; C6 = 19.53 nM
C12 = 555.56 nM; C6 = 9.77 nM
C12 = 185.19 nM; C6 = 5000.0 nM
C12 = 185.19 nM; C6 = 2500.0 nM
C12 = 185.19 nM; C6 = 1250.0 nM
C12 = 185.19 nM; C6 = 625.0 nM
C12 = 185.19 nM; C6 = 312.5 nM
C12 = 185.19 nM; C6 = 156.25 nM
C12 = 185.19 nM; C6 = 78.12 nM
C12 = 185.19 nM; C6 = 39.06 nM
C12 = 185.19 nM; C6 = 19.53 nM
C12 = 185.19 nM; C6 = 9.77 nM
C12 = 61.73 nM; C6 = 5000.0 nM
C12 = 61.73 nM; C6 = 2500.0 nM
C12 = 61.73 nM; C6 = 1250.0 nM
C12 = 61.73 nM; C6 = 625.0 nM
C12 = 61.73 nM; C6 = 312.5 nM
C12 = 61.73 nM; C6 = 156.25 nM
C12 = 61.73 nM; C6 = 78.12 nM
C12 = 61.73 nM; C6 = 39.06 nM
C12 = 61.73 nM; C6 = 19.53 nM
C12 = 61.73 nM; C6 = 9.77 nM
C12 = 20.58 nM; C6 = 5000.0 nM
C12 = 20.58 nM; C6 = 2500.0 nM
C12 = 20.58 nM; C6 = 1250.0 nM
C12 = 20.58 nM; C6 = 625.0 nM
C12 = 20.58 nM; C6 = 312.5 nM
C12 = 20.58 nM; C6 = 156.25 nM
C12 = 20.58 nM; C6 = 78.12 nM
C12 = 20.58 nM; C6 = 39.06 nM
C12 = 20.58 nM; C6 = 19.53 nM
C12 = 20.58 nM; C6 = 9.77 nM
Fluorescence
100000
75000
50000
25000
0
0
6
12
18
24
Time (h)

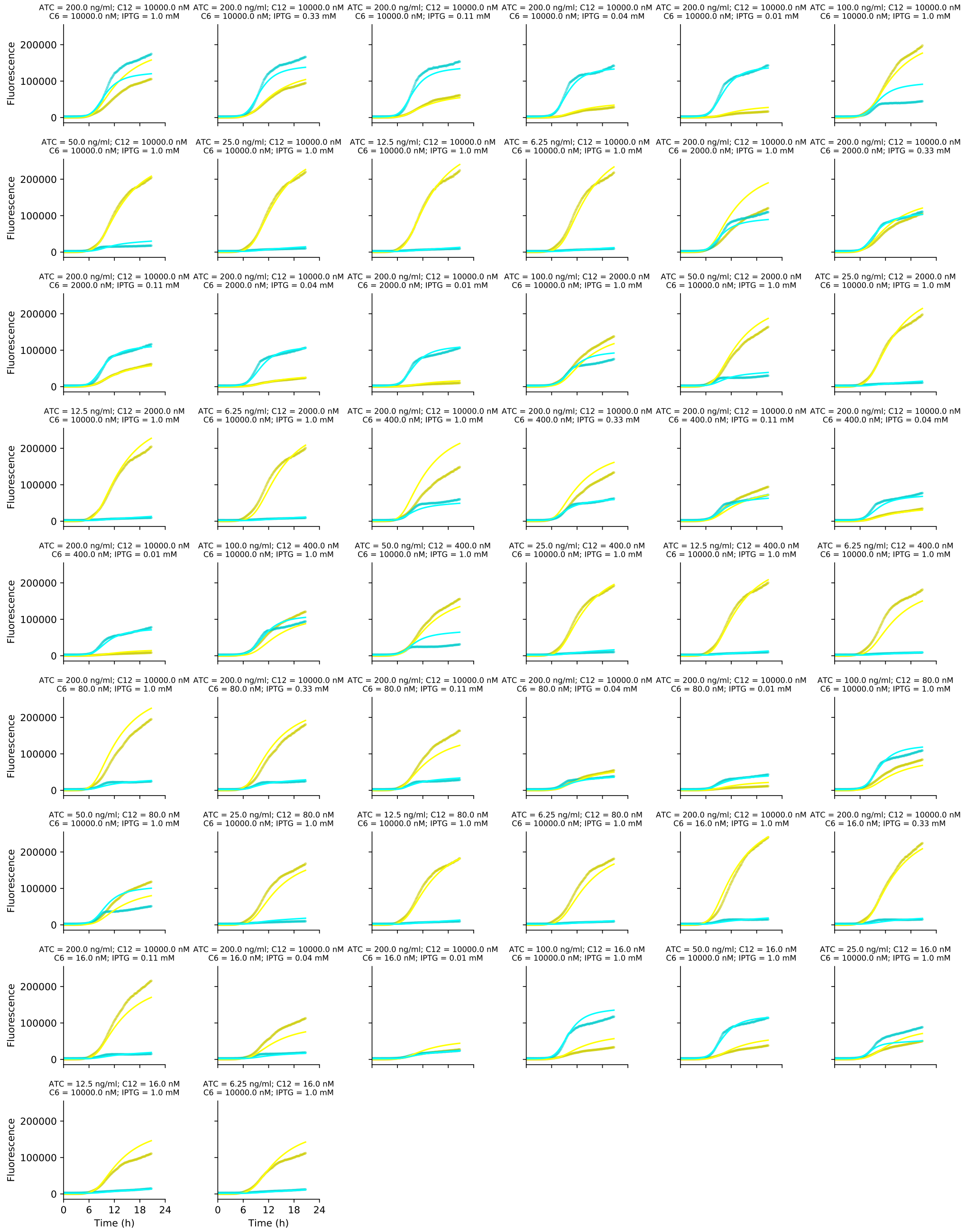

Supplementary Figure 2: Raw timecourse fluorescence traces. . Fluorescence output, measured in microplate fluorometer assays, of the Exclusive Receiver circuit, plotted as CFP (dark cyan) and YFP (dark yellow) fluorescence against time, at the concentrations of C6, C12, ATC, and IPTG indicated. Model simulations (cyan and yellow) of the maximum likelihood parameters are overlaid. Source data are provided as a Source Data file.

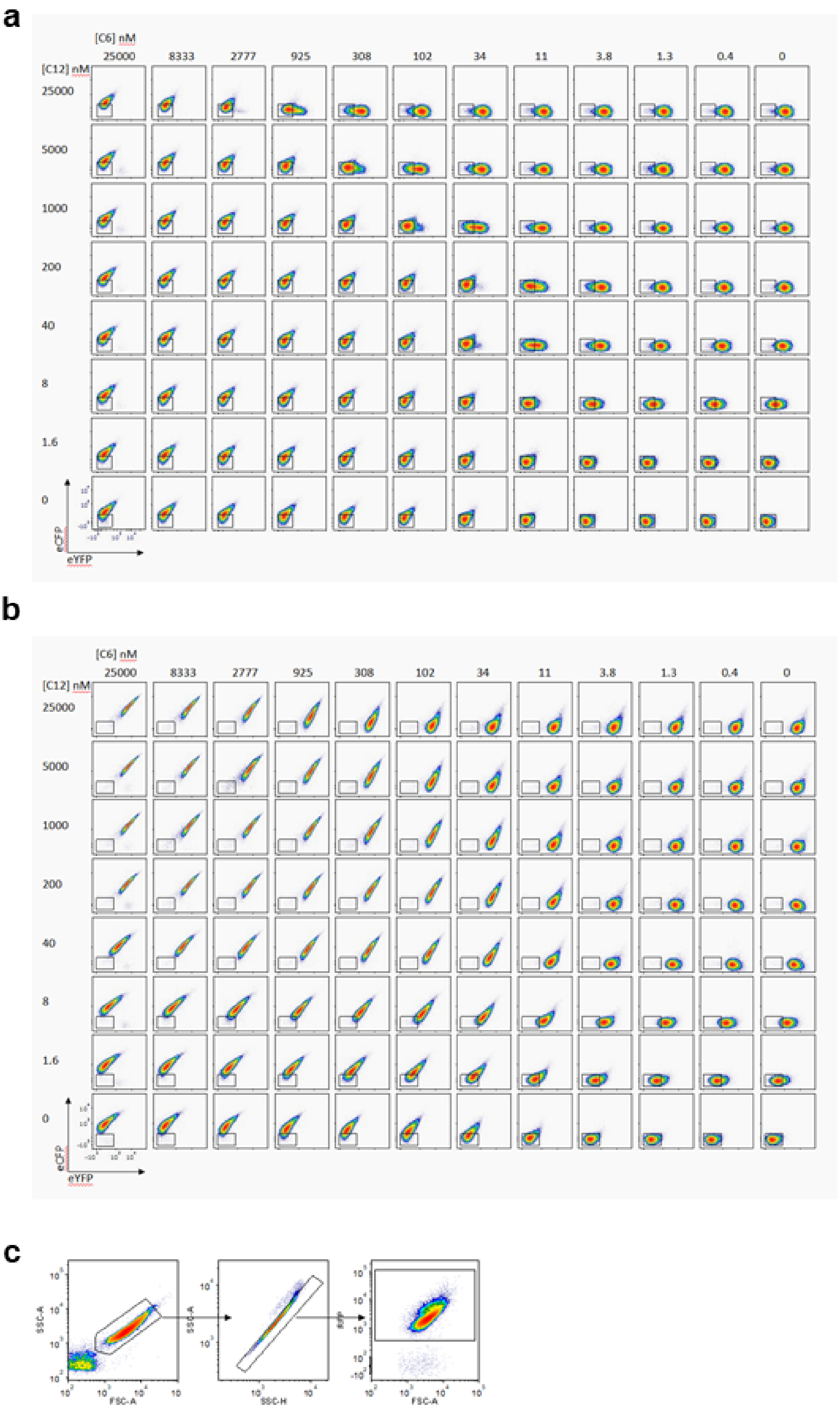

Supplementary Figure 3: Flow cytometry measurements of naive cells a, Exclusive Receiver cells cultured in the indicated concentrations of C6 and C12. YFP fluorescence is plotted on the X-axis while CFP fluorescence is plotted on the Y-axis. b, Receiver cells cultured in the indicated concentrations of C6 and C12. Square indicates the position of untreated cells. c, Gating strategy. Cells also constitutively express mRFP1 via a genomic transgene. Only RFP^{+} cells were used for analysis and electronic noise, cell debris and cell clusters were excluded sequentially. Source data are provided as a Source Data file.

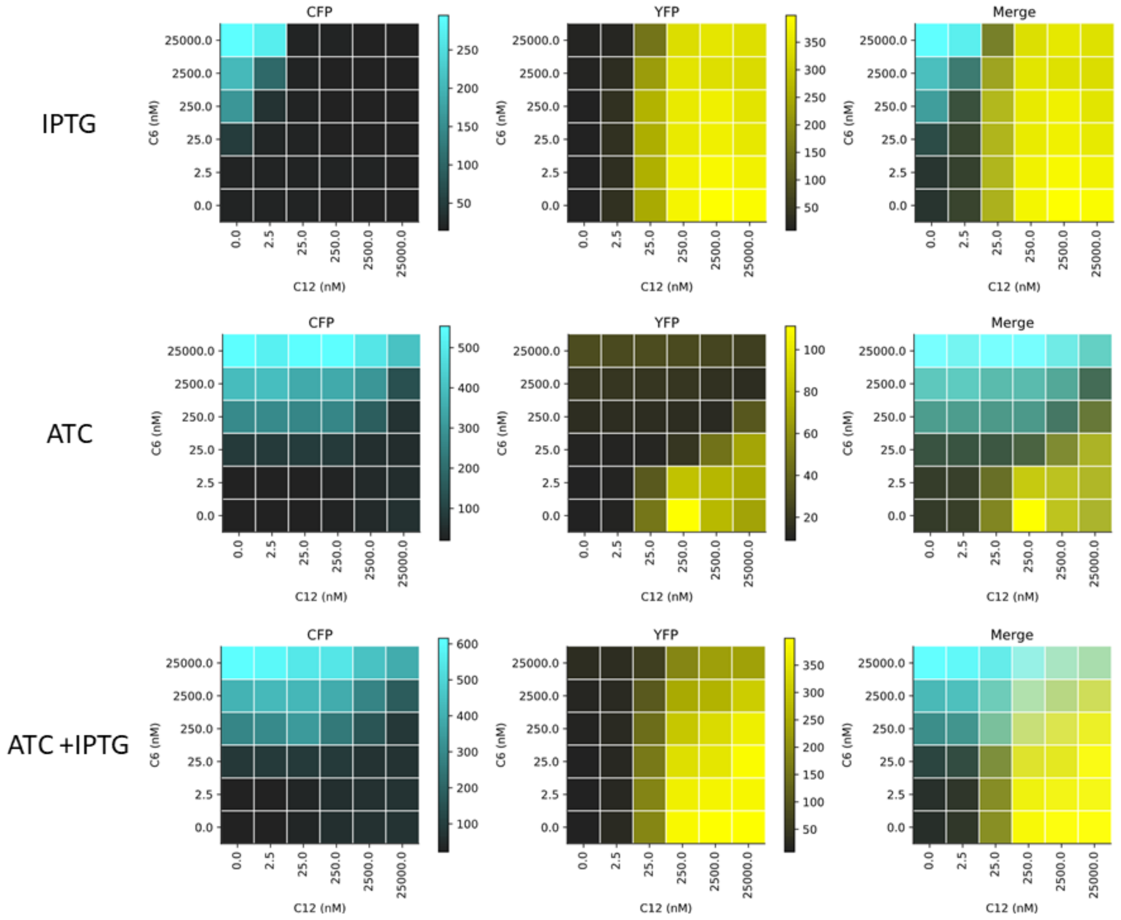

Supplementary Figure 4: ATC and IPTG derepress mutual inhibition. Fluorescence output, measured in microplate fluorometer assays, of the Exclusive Receiver circuit represented as a ratio of CFP- (left) or YFP- (right) fluorescence to RFP fluorescence during exponential phase cultured in the presence of 1 mM IPTG (top), 100 ng/ml ATC (middle) or 1 mM IPTG + 100 ng/ml ATC and the concentrations of 3O-C6-HSL (C6) and 3O-C12-HSL (C12) indicated (see methods for a description of analysis). Source data are provided as a Source Data file.

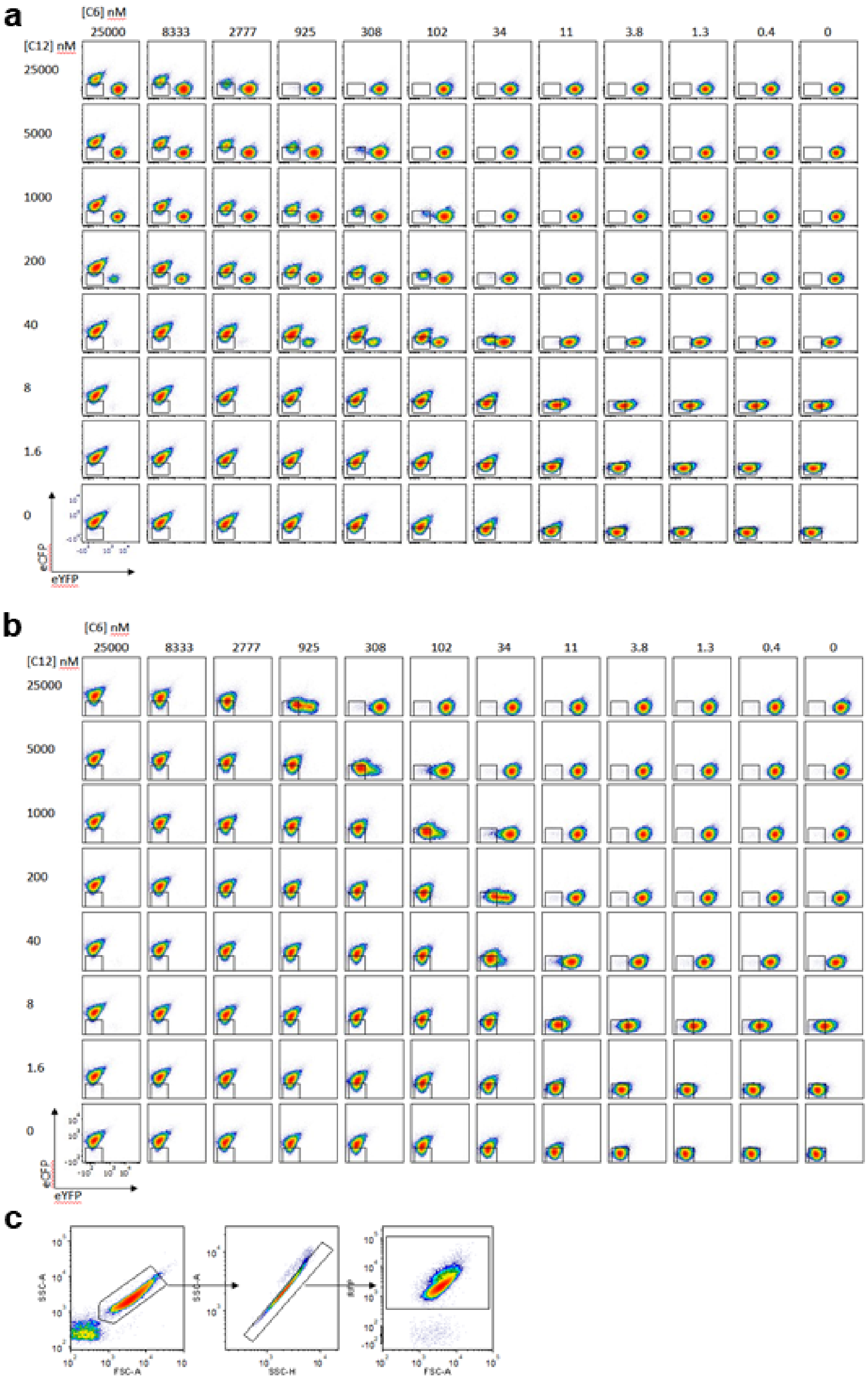

Supplementary Figure 5: Flow cytometry measurements of conditioned cells a, Cells conditioned in 500 nM C12 then exposed to the indicated concentrations of C6 and C12. YFP fluorescence is plotted on the X-axis while CFP fluorescence is plotted on the Y-axis. b, Cells conditioned in 500 nM C6 then exposed to the indicated concentrations of C6 and C12. Square indicates the position of untreated cells. c, Gating strategy. Cells also constitutively express mRFP1 via a genomic transgene. Only RFP^{+} cells were used for analysis and electronic noise, cell debris and cell clusters were excluded sequentially. Source data are provided as a Source Data file.

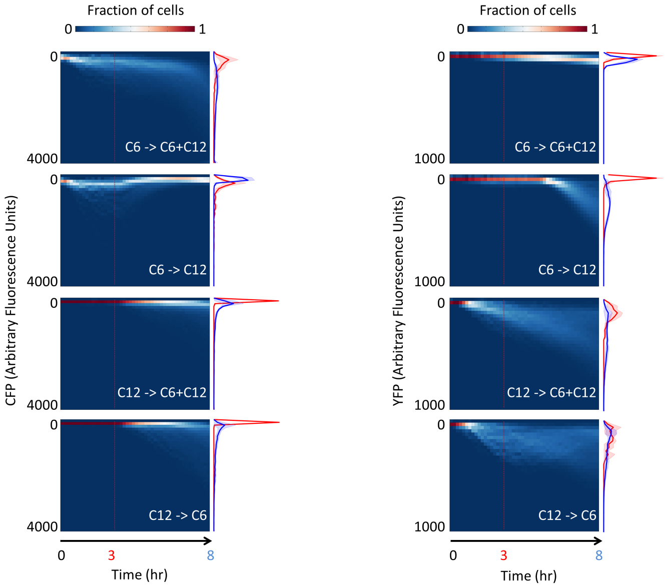

Supplementary Figure 6: Microfluidics measurements of bistability. Single-cell data used to compute ratios in Figure 2c. Cells were grown in microfluidic chips for 3 hours in the presence of either 37 nM C6 (rows 1 and 2) or 100 nM C12 (rows 3 and 4). Then media was changed to 100 nM C12 + 37 nM C6 (rows 1 and 3) or 100 nM C12 (row 2) or 37 nM C6 (row 4) . Cells were imaged with a frame rate of (1 frame/10 minutes). Left panels in each column are kymographs of the CFP (left column) or YFP (right column) expression per-cell, and fraction of cells as a heat map. Histograms represent the populations at 3 hours (red) and 8 hours (blue). Lines and shaded region represent the mean and standard deviation, respectively, over n = 4 biological replicates performed over 4 different days.

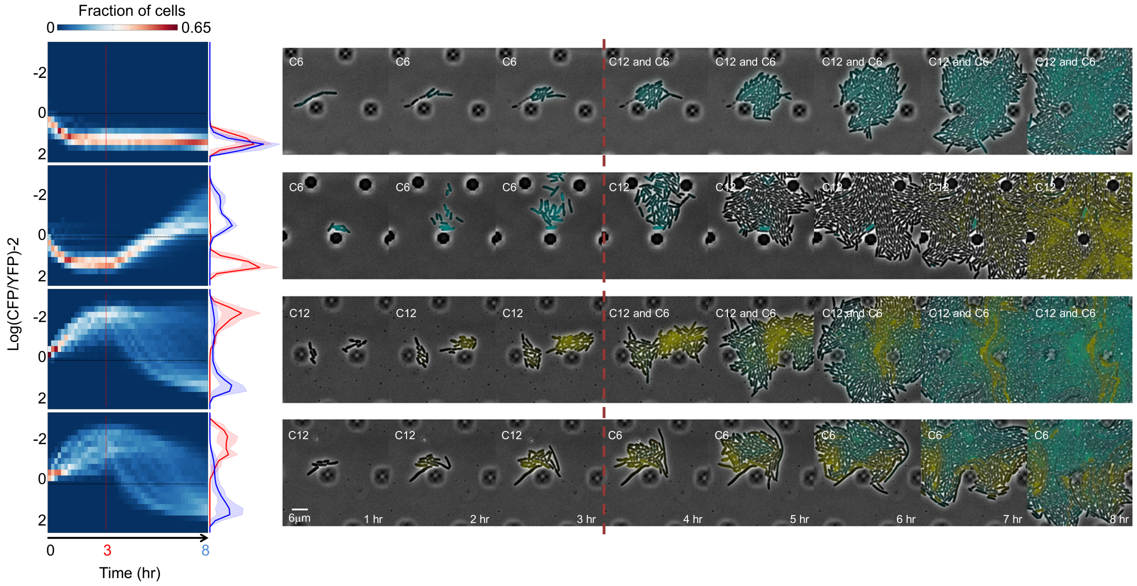

Supplementary Figure 7: Bistability and switching of single cells is robust to high C6 signal concentration. Cells were grown in microfluidic chips for 3 hours in the presence of either 1 μM C6 (rows 1 and 2) or 100 nM C12 (rows 3 and 4). Then media was changed to 100 nM C12 + 1 μM C6 (rows 1 and 3) or 100 nM C12 (row 2) or 1 μM C6 (row 4). Cells were imaged with a frame rate of (1 frame/10 minutes). Left panels are kymographs of the log-ratio of CFP expression per-cell to YFP expression per-cell, and fraction of cells as a heat map. Histograms represent the populations at 3 hours (red) and 8 hours (blue). Lines and shaded region represent the mean and standard deviation, respectively, n = 4 biological replicates performed over 4 different days. Right panels are sample montages of cells switching state (rows 3 and 4) or exhibiting bistablity (rows 1 and 2); phase contrast and fluorescence channel ranges chosen for display.

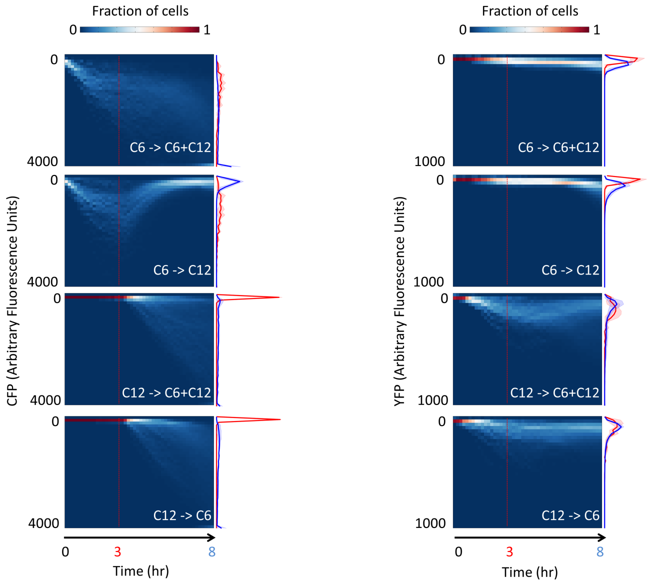

Supplementary Figure 8: Microfluidics measurements of bistability with high C6 signal concentration. Single-cell data used to compute ratios in 7. Cells were grown in microfluidic chips for 3 hours in the presence of either 1 μM C6 (rows 1 and 2) or 100 nM C12 (rows 3 and 4). Then media was changed to 100 nM C12 + 1 μM C6 (rows 1 and 3) or 100 nM C12 (row 2) or 1 μM C6 (row 4) . Cells were imaged with a frame rate of (1 frame/10 minutes). Left panels in each column are kymographs of the CFP (left column) or YFP (right column) expression per-cell, and fraction of cells as a heat map. Histograms represent the populations at 3 hours (red) and 8 hours (blue). Lines and shaded region represent the mean and standard deviation, respectively, over n = 4 biological replicates performed over 4 different days.

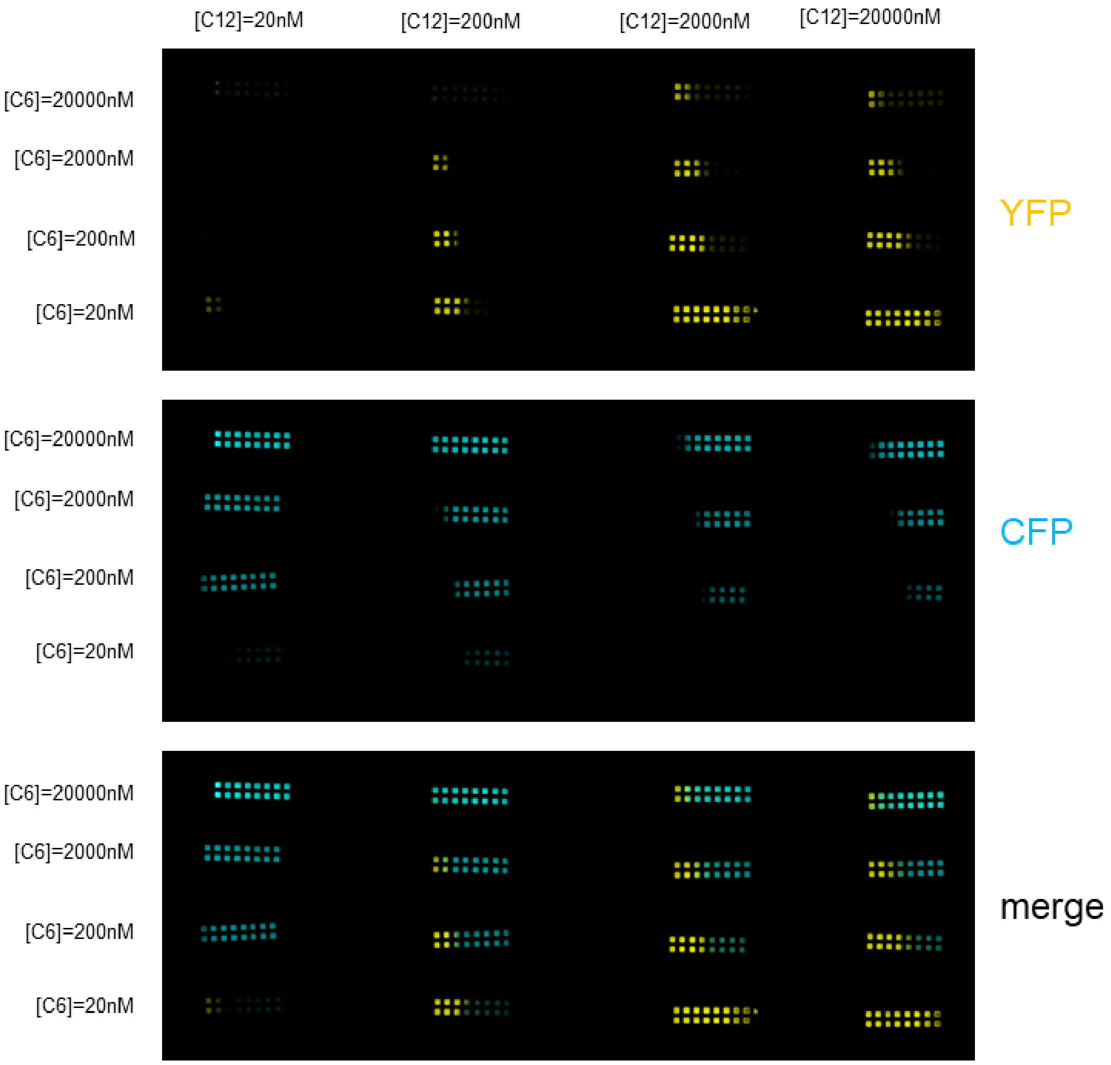

Supplementary Figure 9: Boundaries summarized in Figure 3c Endpoint fluorescence microscopy of Exclusive Receiver cells grown in transient gradients of signals (C12 diffusing from the left, C6 diffusing from the right) at the spatial average concentrations indicated and in the context of 10 μM IPTG throughout.

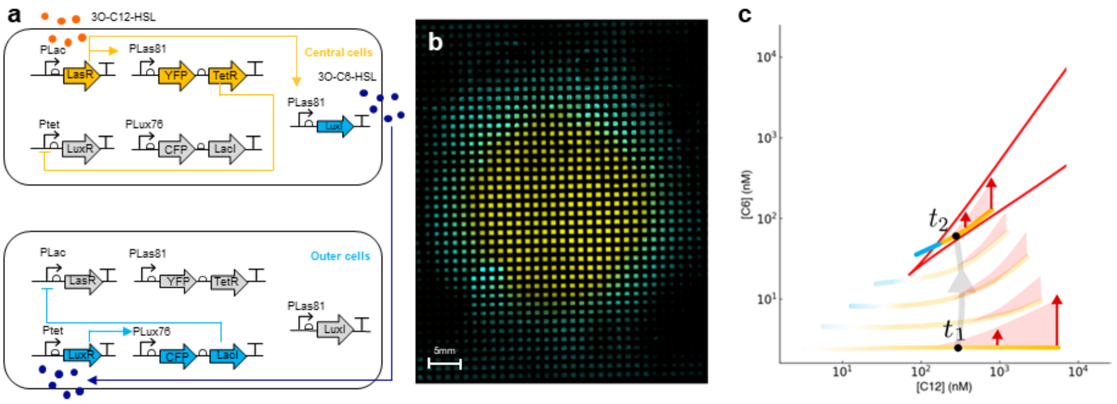

Supplementary Figure 10: Swapping primary and secondary morphogens also produces patterning. a, A circuit diagram of exclusive reporter cells co-transformed with the P81-LuxI relay device that responds to C12 by producing C6. Cells that experience high levels of C12 (central cells) will express YFP, TetR, and LuxI, causing them to produce C6 but be unable to sense it. Neighbouring cells (outer cells) that do not experience C12 will sense C6 and express CFP and LacI. **b**, Isogenic cells transformed with the circuit shown in (a) grown for 24 hours in the presence of a gradient of C12 diffusing from the centre express CFP and YFP in mutually exclusive domains of gene expression.**c**, A simulation in C6-C12 space over time (t1-t2) labelling points in physical space by their CFP and YFP expression (cyan and yellow points), and showing the production of C6 as vectors (red arrows) that move the spatial average (black point) toward increasing C6. The bistable region is outlined in red.

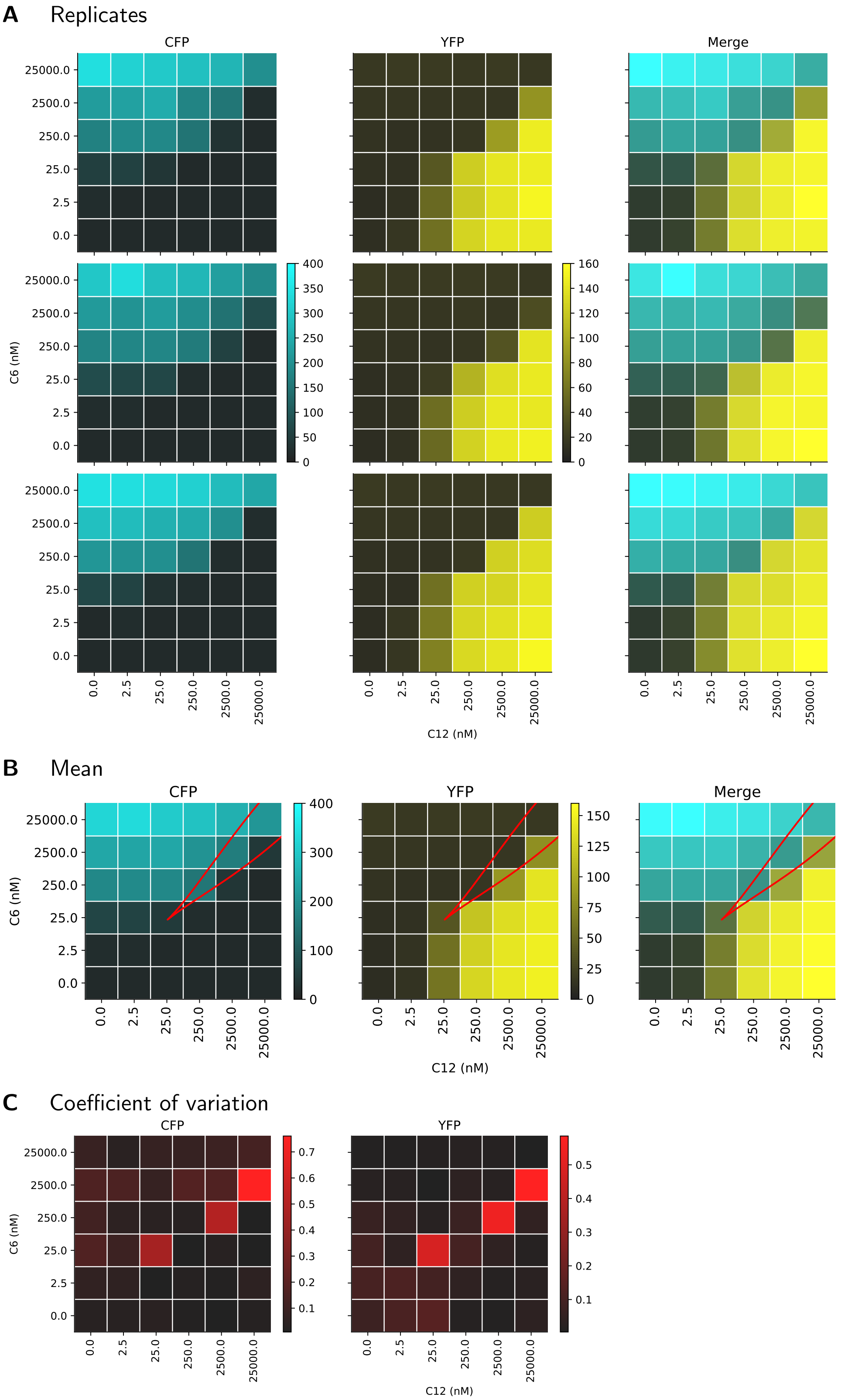

Supplementary Figure 11: Variability in Exclusive Receiver circuit characterization. Ratiometric characterization [1, 2] was applied to time-series measurements of CFP, YFP and RFP fluorescence in cells expressing the Exclusive Receiver circuit. (**A**) Three independent replicates are shown on an equivalent colour scale that reports ratiometric promoter activity of CFP in response to 3O-C6-HSL and YFP in response to 3O-C12-HSL. In both cases, chromosomally expressed mRFP1 are used as ratiometric controls. Also shown are the mean (**B**) and coefficient of variation (**C**) of each condition across these replicates. In B, the region of bistability described in Section 2.2 is indicated by the red line. Source data are provided as a Source Data file.

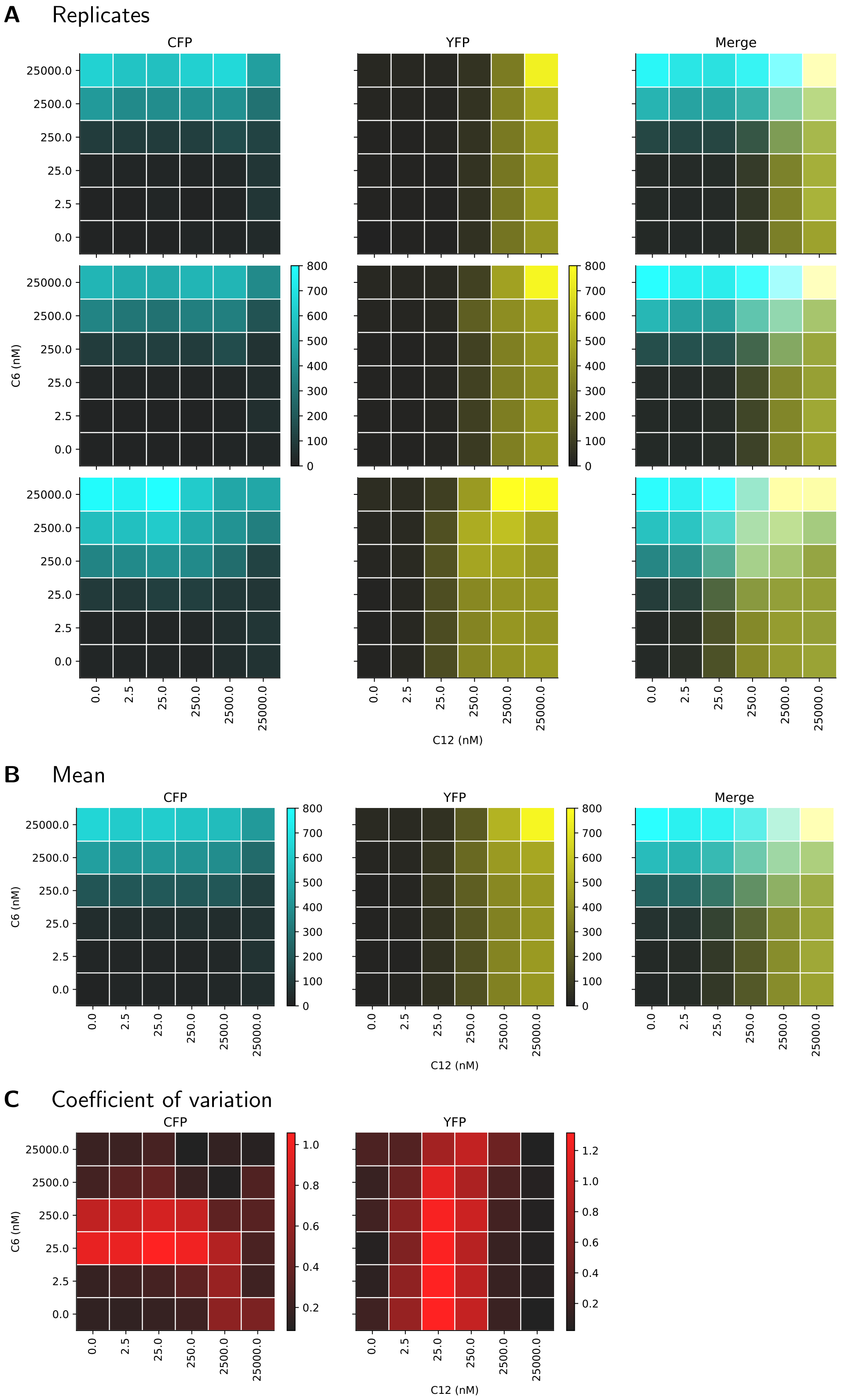

Supplementary Figure 12: Variability in Receiver circuit characterization. Ratiometric characterization [1, 2] was applied to time-series measurements of CFP, YFP and RFP fluorescence in cells expressing the Receiver circuit. (**A**) Three independent replicates are shown on an equivalent colour scale that reports ratiometric promoter activity of CFP in response to 3O-C6-HSL and YFP in response to 3O-C12-HSL. In both cases, chromosomally expressed mRFP1 are used as ratiometric controls. Also shown are the mean (**B**) and coefficient of variation (**C**) of each condition across these replicates. Source data are provided as a Source Data file.

Replicate **A**

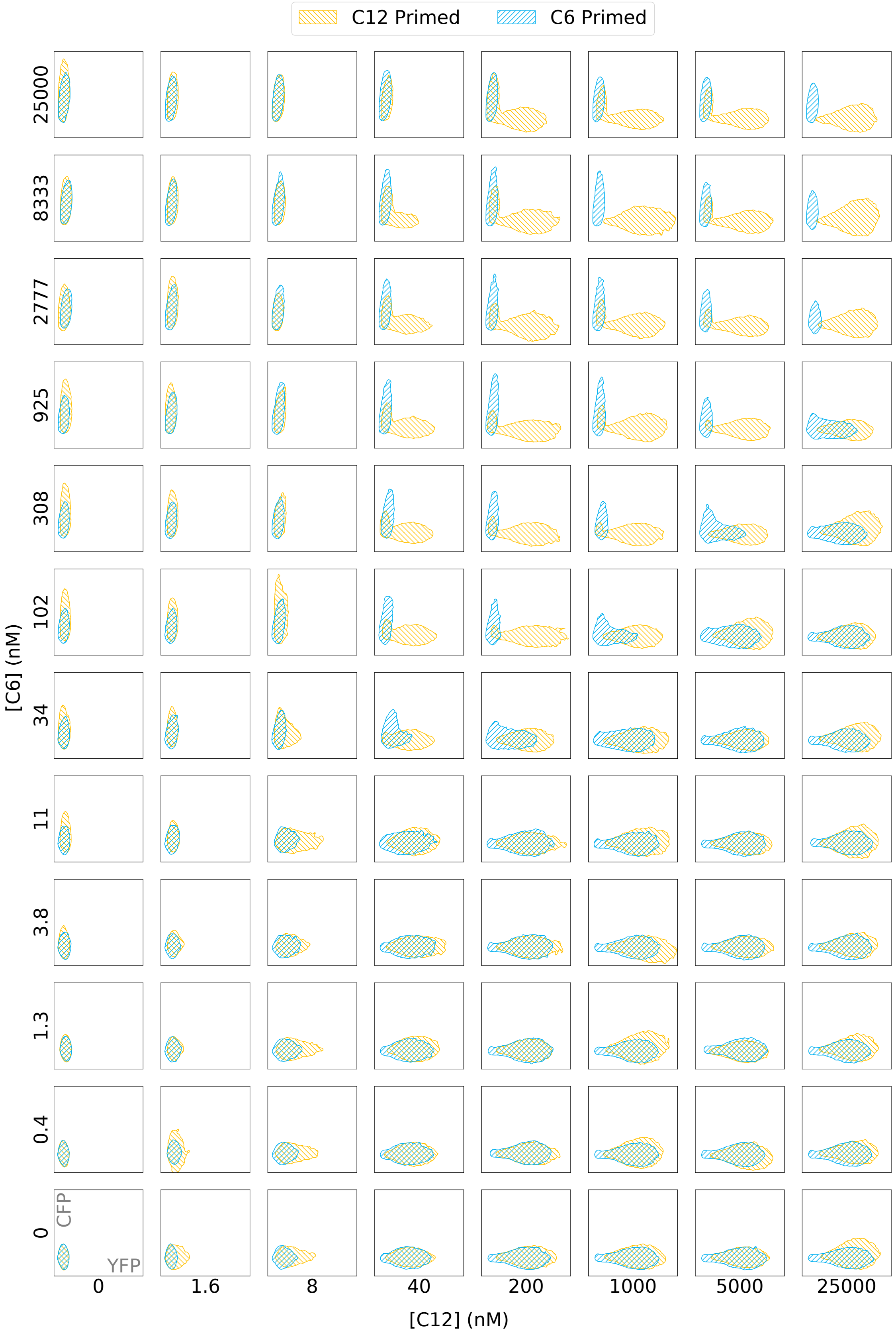

C12 Primed
C6 Primed
25000
8333
2777
925
308
102
34
11
3.8
1.3
0.4
0
[C6] (nM)
CFP
YFP
0
1.6
8
40
200
1000
5000
25000
[C12] (nM)

Replicate **B**

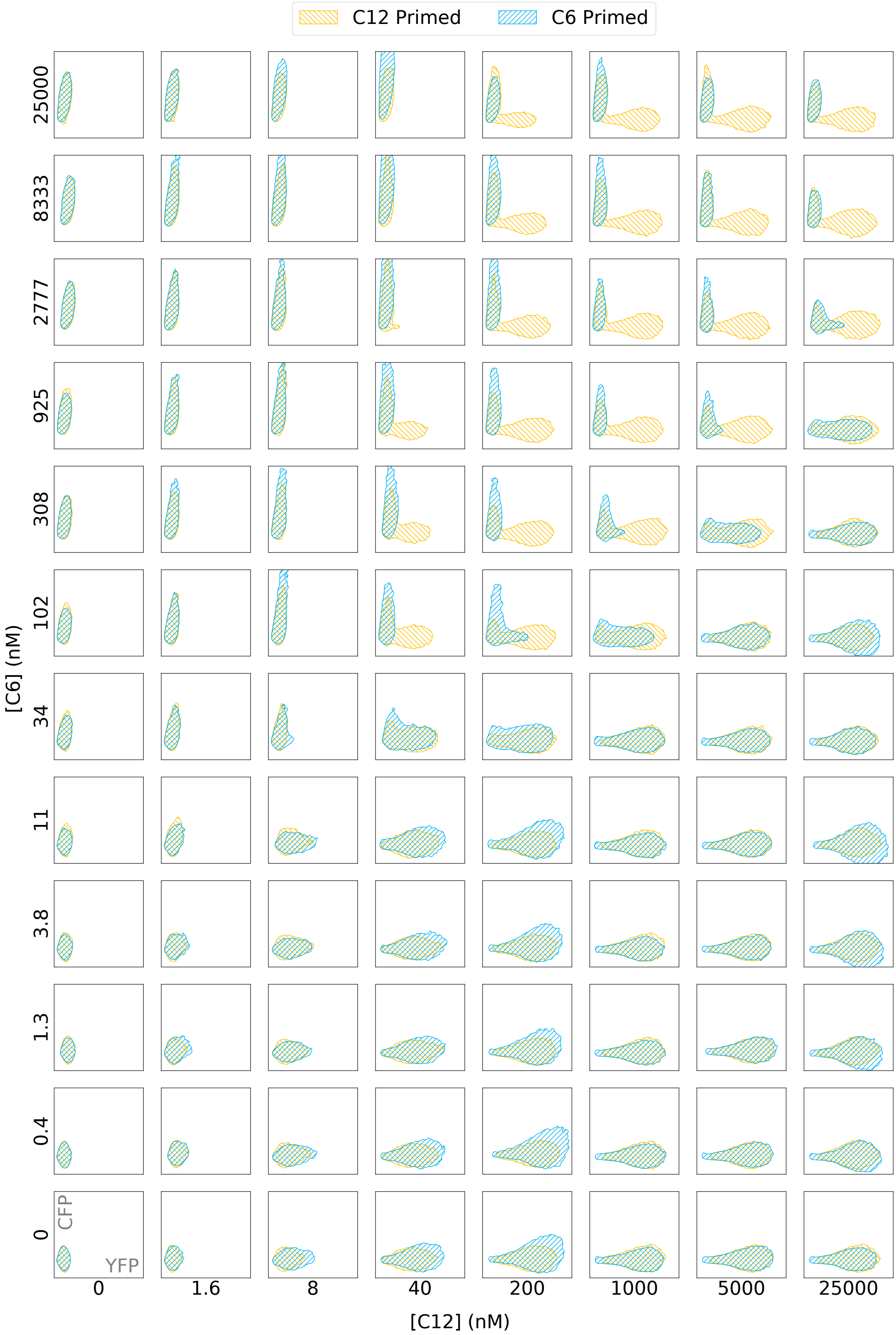
C12 Primed
C6 Primed
25000
8333
2777
925
308
102
34
11
3.8
1.3
0.4
0
[C6] (nM)
CFP
YFP
0
1.6
8
40
200
1000
5000
25000
[C12] (nM)

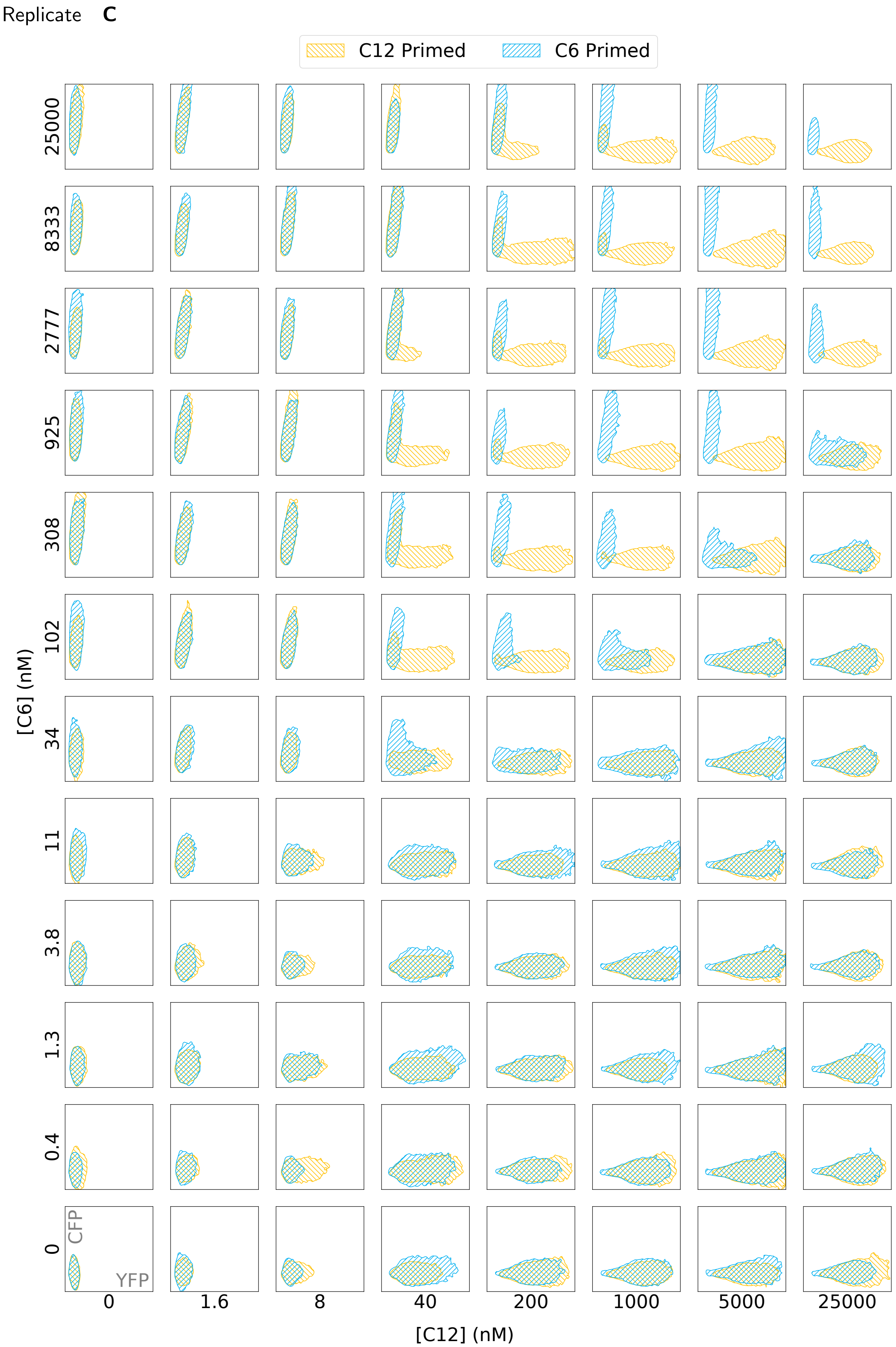

Supplementary Figure 13: Hysteresis flow cytometry experiments. Each replicate (**A-C**) is the overlay of two independent experiments with cells initialised in either 500 nM 3O-C6-HSL or 500 nM 3O-C12-HSL and transferred into varying concentrations of morphogens as indicated on the major axes. Regions of concentrations where the final population state depends on its initial state are visible along the diagonal. CFP and YFP fluorescence on the minor axes are normalised by RFP and centred such that auto-fluorescence of the 0 nM dilution is fixed in the bottom left. Source data are provided as a Source Data file.

2 Supplementary Methods

Supplementary Table 1: Primers used for Gibson assembly of the exclusive receiver circuit

Primer name	Sequence
PGMSR014 LacIND F	TTTATTTGATGCCTGGCTTTATTACTGCCCGCTTTCCAGTCGG
PGMSR015 LacIND R	GCGGGCAGTAATAAAGCCAGGCATCAAATAAAACGAAAGG
PGMSR038 LacI300 F	TTTAGGACTGGACGGCGAAGGCCTGATGGAGTTCTCTAGTAT-TATTACTTGTACAGCTCGTCCATGCC
PGMSR039 LacI300 R	AGAACTCCATCAGGCCTTCGCCGTCCAGTCCTAAAATGGT-GAATGTGAAACCAGTAACGTTAT
PG081 Kan F	GGAATCGAATGCAACCGGC
PG082 Kan R	TGATGCGCTGGCAGTGTTC
PG342 Tet33 F	TCACACAGGACTACTAGATGTCCAGATTAGATAAAAG-TAAAGTGATTAACAGCGC
PG343 Tet33 R	ATCTAATCTGGACATCTAGTAGTCCTGTGTGATTATTACTTG-TACAGCTCGTCCATGCCG
PG373 TetAAV F	CGAAAACTACGCTGCTGCTGTTTAATAACCAGGCAT-CAAATAAAACGAAAGGC
PG374 TetAAV R	TGGTTATTAAACAGCAGCAGCGTAGTTTTCGTCGTTTGCTGCG-GACCCAC
JS3F Ori	GAGTGTATACTGGCTTACTATGTTGGCACTG
JS3R Ori	CAGTGCCAACATAGTAAGCCAGTATACACTC

2.1 Differential Equation Models and Parameter Inference

In this section, we derive ordinary differential equation (ODE) models for the reaction kinetics underlying the Exclusive Receiver circuit. These derivations broadly follow the derivations of the Receiver circuit in [3] and [4]. Importantly, we introduce differences in that original derivation that lead to changes in the location of bifurcations in (C12,C6) space, when those derivations are extended to incorporate dynamics of the repressor proteins LacI and TetR, and their chemical inhibitors IPTG and ATC.

Dynamic characterization with inference graphs

In order to infer the parameters of the Exclusive Receiver circuit, we adopt the strategy described in [4], evaluating parameters of sub-circuits first, and propagating their inferred values to larger circuits that embed those same parameters. This results in an inference graph, where we infer parameters over a sequence of models and corresponding datasets.

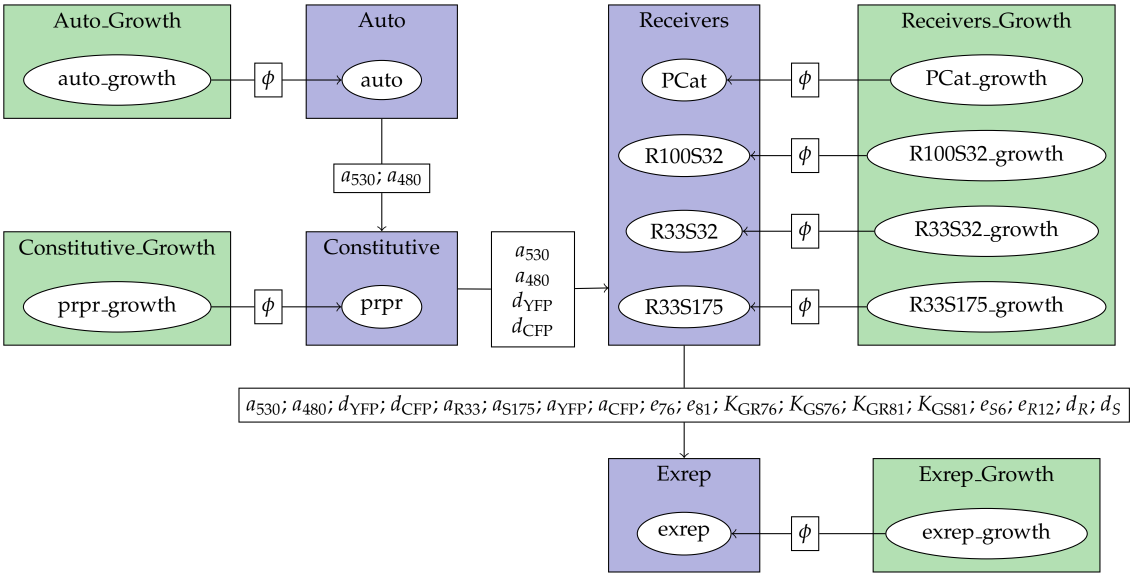

Supplementary Figure 14: Inference graph for characterizing the Exclusive Receiver circuit. Blue rectangular nodes are inference problems for collections of synthetic gene circuit models, compared with CFP and YFP measurements, while green rectangular nodes are inference problems for the growth models of the circuits in the downstream blue nodes, compared with OD_{600} measurements. Internal to the coloured nodes are white elliptical nodes, which correspond to individual synthetic gene circuits and one or more associated dataset(s). White rectangular nodes are the sets of inferred parameters that are propagated between nodes. To simplify the notation, ϕ has been used to denote the set of growth model parameters, which are culture-specific (local) values for r, K and t_{lag}, and are propagated as maximum likelihood estimates. All other parameters are global (non-culture-specific) values and propagated as marginal posterior estimates.

The simplest possible cell line to characterize is one in which there is no synthetic gene circuit at all. Applying dynamic characterization in this context enables us to quantify autofluorescence, and so we name this circuit *auto*. Therefore, we measured cells under a range of conditions to explore how gene expression capacity influenced time-series measurements at fluorescence wavelengths corresponding to CFP and YFP. Subsequently, we characterize a circuit (prpr) in which CFP and YFP are driven by constitutive promoters (PR), enabling us to characterize the rates of degradation of the fluorescent proteins. Next, we use four variants of simple HSL receiver (PCat, R100S32, R33S32 and R33S175) to characterize the genetic parts associated with LuxR and LasR receiver proteins and their interactions with HSL molecules 3OC6HSL (C6) and 3OC12HSL (C12). Finally, having obtained parameter estimates for parts associated with CFP, YFP, LuxR and LasR, we characterize the Exclusive Receiver circuit (exrep) itself, establishing quantitative estimates for the parts associated with TetR and LacI repressor proteins.

In the following, we introduce the models for each of the circuits just mentioned, define their parameters and present results of the inference.

Autofluorescence model

The model we used for autofluorescence assumes that the rate of autofluorescence is constant, and that the fluorescent material dilutes with cell growth. As such, the equations for intracellular autofluorescence corresponding to CFP and YFP are

$$\frac{d\rho}{dt} = \gamma(\rho).\rho \tag{1a}$$

$$\frac{dc_{480}}{dt} = a_{480} - \gamma(\rho).c_{480} \tag{1b}$$

$$\frac{dc_{530}}{dt} = a_{530} - \gamma(\rho).c_{530} \tag{1c}$$

where $\gamma(\rho) = r(1 - \frac{\rho}{K})$ is the specific growth rate of the cell culture with density ρ.

To compare with experimental measurements, we consider the bulk fluorescence given by

$$B_{480} = \rho.c_{480} + b_{480} \tag{2a}$$

$$B_{530} = \rho.c_{530} + b_{530} \tag{2b}$$

where c_{480} and c_{530} are modelled as in (1). Here, the quantities b_{480} and b_{530} represent background fluorescence at 480 nm and 530 nm, corresponding to CFP and YFP respectively.

Inference. We use the data in [4] to infer the parameters of the auto circuit. Cells were treated with EtOH to perturb cell growth, enabling us to determine how autofluorescence changes with different cellular growth rates. The priors used are detailed in the following table.

Supplementary Table 2: Priors for the auto circuit characterization. In the unit column, Fl denotes fluorescence units. The scaling column refers to the proposal distribution used to generate new parameters during MCMC.

Parameter	Description	Unit	Distribution	Scaling
a_{480}	Autofluorescence (CFP)	Fl.cell^{-1}.h^{-1}	$U(10^{-3}, 10^{3})$	Log
a_{530}	Autofluorescence (YFP)	Fl.cell^{-1}.h^{-1}	$U(10^{-3}, 10^{3})$	Log
b_{480}	Background fluor. (CFP)	Fl	$U(0, 10^{4})$	Real
b_{530}	Background fluor. (YFP)	Fl	$U(0, 5 \times 10^{3})$	Real

The marginal posterior estimates are shown in Figure 15. Simulation of the maximum likelihood estimate is shown in Figure 16.

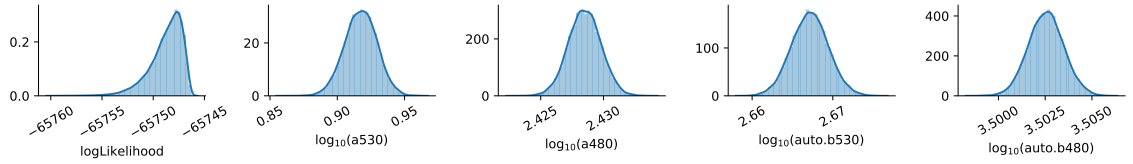

Supplementary Figure 15: Marginal parameter posterior estimates of the auto model parameters. The marginal distributions are computed from 20 independent MCMC chains.

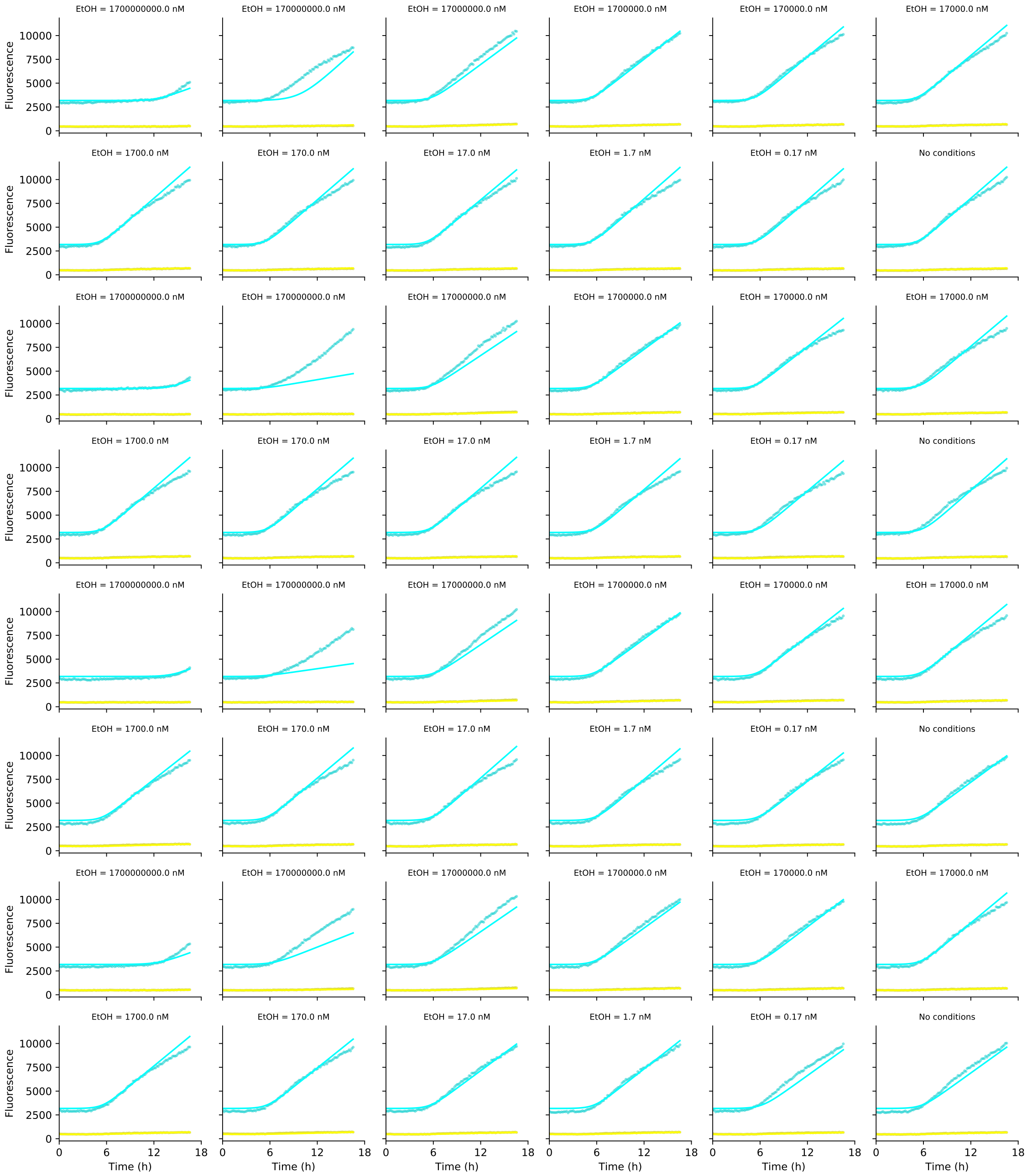

Supplementary Figure 16: Comparison of auto model with fluorescence measurements. Simulations are for B_{480} (cyan lines) and B_{530} (yellow lines), evaluated with the maximum likelihood parameter set. Measurements of bulk culture fluorescence are shown for the emission wavelengths corresponding to CFP (cyan circles) and YFP (yellow circles). EtOH treatment concentrations are indicated atop each panel.

Constitutive (prpr) model

The prpr circuit described above uses the constitutive PR promoter to drive CFP and YFP expression, in two separate operons. Following the derivation in [4], we arrive at a system of equations that describe the time-evolution of the intracellular concentrations of CFP and YFP as

$$\frac{dc}{dt} = \gamma(\rho).c \tag{3a}$$

$$\frac{dc_{\mathrm{CFP}}}{dt} = a_{\mathrm{CFP}} - (d_{\mathrm{CFP}} + \gamma(\rho))c_{\mathrm{CFP}} \tag{3b}$$

$$\frac{dc_{\mathrm{YFP}}}{dt} = a_{\mathrm{YFP}} - (d_{\mathrm{YFP}} + \gamma(\rho))c_{\mathrm{YFP}} \tag{3c}$$

where a_{CFP} and a_{YFP} are aggregated parameters that incorporate the rate of transcription and translation of CFP and YFP respectively. To compare with bulk culture fluorescence data, we use the observer model

$$B_{480} = \rho.(c_{\mathrm{CFP}} + c_{480}) + b_{480} \tag{4a}$$

$$B_{530} = \rho.(c_{\mathrm{YFP}} + c_{530}) + b_{530} \tag{4b}$$

where the dynamics of c_{480} and c_{530} are governed by equations (1) above.

Inference. We use the data in [4] to infer the parameters of the prpr circuit. Cells were treated with chloramphenicol to perturb cell growth, enabling us to establish how constitutively expressed proteins are altered with different cellular growth rates. While the model above does not explicitly describe any explicit functional response to chloramphenicol, our general strategy of allowing the cell growth parameters to vary across different measurements enables the effect of chloramphenicol on growth rates to be implicitly captured. The quantification of autofluorescence was reused from the auto circuit (upstream in the inference graph), but the background fluorescence parameters were re-inferred. The priors used are detailed in Table 3. The marginal posterior estimates are shown in Figure 17.

Supplementary Table 3: Priors for the prpr circuit characterization. In the unit column, Fl denotes fluorescence units. The scaling column refers to the proposal distribution used to generate new parameters during MCMC.

Parameter	Description	Unit	Distribution	Scaling
d_{CFP}	CFP degradation	h^{-1}	$U(10^{-3}, 10^{0})$	Log
d_{YFP}	YFP degradation	h^{-1}	$U(10^{-3}, 10^{0})$	Log
a_{CFP}	CFP synthesis	$\mathrm{Fl.cell}^{-1}.\mathrm{h}^{-1}$	$U(10^{0}, 10^{5})$	Log
a_{YFP}	YFP synthesis	$\mathrm{Fl.cell}^{-1}.\mathrm{h}^{-1}$	$U(10^{0}, 10^{5})$	Log
b_{480}	Background fluor. (CFP)	Fl	$U(0, 10^{4})$	Real
b_{530}	Background fluor. (YFP)	Fl	$U(0, 5 \times 10^{3})$	Real

Simulation of the maximum likelihood estimate is shown in Figure 18. The simulated CFP and YFP largely agree with the measured fluorescence at the culture level. As the effect of chloramphenicol is not explicitly modelled here, this comparison indicates that the majority of the effect of chloramphenicol can be described via its effect on cell growth. Any additional direct effect on CFP and YFP expression directly, is relatively minor.

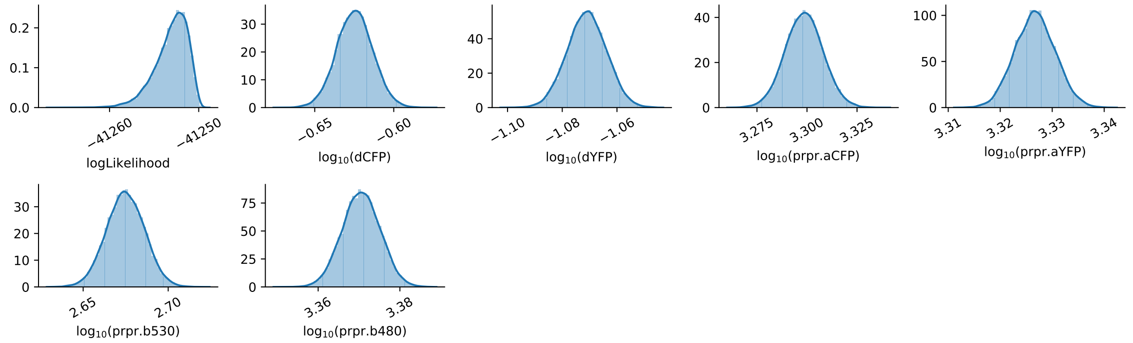

Supplementary Figure 17: Marginal parameter posterior estimates of the prpr model parameters. The marginal distributions are computed from 20 independent MCMC chains.

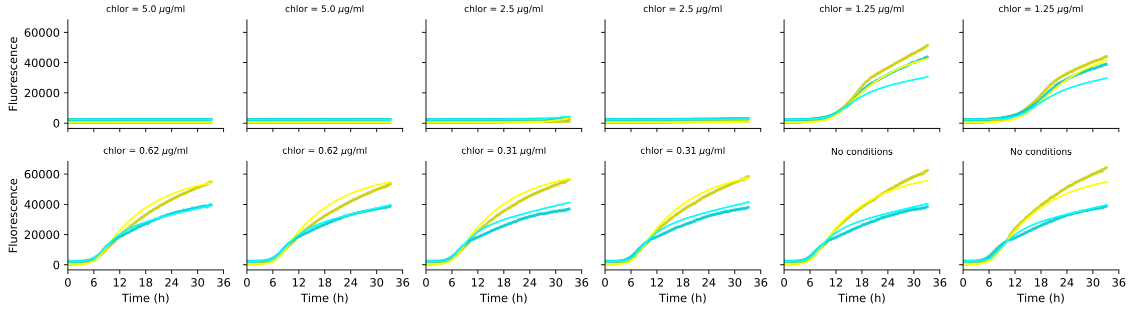

Supplementary Figure 18: Comparison of prpr model with fluorescence measurements. Simulations are of bulk CFP (cyan lines) and YFP (yellow lines) fluorescence, evaluated with the maximum likelihood parameter set. Measurements of bulk culture fluorescence are shown in for CFP (cyan circles) and YFP (yellow circles). Chloramphenicol treatment concentrations are indicated atop each panel.

Receiver model

We consider the dynamic characterization of the HSL Receiver circuit introduced in [3] and modelled dynamically in [4, 5]. In this circuit, which we refer to in the main text as the Receiver, two variations of the wild-type PLux promoter, PLux76 and PLas81, were engineered to bind preferentially to activated LuxR and LasR complexes respectively. As LuxR favours binding of C6 and LasR favours binding of C12, optimized expression of LuxR and LasR can lead to near-orthogonal intracellular detection of C6 and C12. The Receiver device was originally measured with PLux76 upstream of the coding sequence for CFP, and PLas81 upstream of the coding sequence for YFP.

Version 1 - Uniform degradation. The first version of the model we introduce is derived in [4]. It is based on the assumption that all degradation processes are of the same order, e.g. LuxR monomers are degraded at a similar rate as LuxR Dimers bound to HSLs. Subsequently, we will introduce an alternative derivation where we assume that complexes are protected from degradation, i.e. that degradation mainly occurs on the monomer level. We start by repeating some of the derivation from [4].

We denote by C_k the HSL molecule with length k carbon chain, and by G_i the PLux76 and PLas81 promoters. Then similar to the derivation in [3], we can specify all of the reactions between the HSLs, LuxR and LasR, and eventual binding of transcriptional regulators to PLux76/PLas81.

$$\begin{aligned}
\text{LuxR} + \text{C}_k &\underset{u_{Rk}}{\overset{b_{Rk}}{\rightleftharpoons}} \text{LuxR-C}_k \\
\text{LasR} + \text{C}_k &\underset{u_{Sk}}{\overset{b_{Sk}}{\rightleftharpoons}} \text{LasR-C}_k \\
\text{LuxR-C}_k + \text{LuxR-C}_k &\underset{u_{Dk}}{\overset{b_{Dk}}{\rightleftharpoons}} \text{LuxR-C}_k\text{-LuxR-C}_k \\
\text{LasR-C}_k + \text{LasR-C}_k &\underset{u_{Ek}}{\overset{b_{Ek}}{\rightleftharpoons}} \text{LasR-C}_k\text{-LasR-C}_k \\
G_i + \text{LuxR-C}_k\text{-LuxR-C}_k &\underset{u_{GiDk}}{\overset{b_{GiDk}}{\rightleftharpoons}} G_i\text{-LuxR-C}_k\text{-LuxR-C}_k \\
G_i + \text{LasR-C}_k\text{-LasR-C}_k &\underset{u_{GiEk}}{\overset{b_{GiEk}}{\rightleftharpoons}} G_i\text{-LasR-C}_k\text{-LasR-C}_k
\end{aligned} \tag{5}$$

Constitutive expression of LuxR and LasR is described by

$$\varnothing \xrightarrow{a_R} \text{LuxR} \qquad \varnothing \xrightarrow{a_S} \text{LasR} \tag{6}$$

Degradation of LuxR and LasR is described by

$$\begin{aligned}
\text{LuxR} &\xrightarrow{d_R} \varnothing & \text{LasR} &\xrightarrow{d_S} \varnothing \\
\text{LuxR-C}_k &\xrightarrow{d_R^1} \text{C}_k & \text{LasR-C}_k &\xrightarrow{d_S^1} \text{C}_k \\
\text{LuxR-C}_k\text{-LuxR-C}_k &\xrightarrow{d_R^2} 2\text{C}_k & \text{LasR-C}_k\text{-LasR-C}_k &\xrightarrow{d_S^2} 2\text{C}_k
\end{aligned} \tag{7}$$

where d_R^1 and d_R^2 are distinguishable from d_R to describe the effect of HSL molecules protecting receiver proteins from degradation.

Inducible expression of CFP and YFP by P_{OLux} and P_{OLas} respectively is described by

$$\begin{aligned}
G_{\text{pLux76}} &\xrightarrow{a0_{\text{pLux76}}} G_{\text{pLux76}} + \text{CFP} \\
G_{\text{pLux76}}\text{-LuxR-C}_k\text{-LuxR-C}_k &\xrightarrow{a1_{\text{pLux76}}} G_{\text{pLux76}}\text{-LuxR-C}_k\text{-LuxR-C}_k + \text{CFP} \\
G_{\text{pLux76}}\text{-LasR-C}_k\text{-LasR-C}_k &\xrightarrow{a1_{\text{pLux76}}} G_{\text{pLux76}}\text{-LasR-C}_k\text{-LasR-C}_k + \text{CFP} \\
G_{\text{pLas81}} &\xrightarrow{a0_{\text{pLas81}}} G_{\text{pLas81}} + \text{YFP} \\
G_{\text{pLas81}}\text{-LuxR-C}_k\text{-LuxR-C}_k &\xrightarrow{a0_{\text{pLas81}}} G_{\text{pLas81}}\text{-LuxR-C}_k\text{-LuxR-C}_k + \text{YFP} \\
G_{\text{pLas81}}\text{-LasR-C}_k\text{-LasR-C}_k &\xrightarrow{a1_{\text{pLas81}}} G_{\text{pLas81}}\text{-LasR-C}_k\text{-LasR-C}_k + \text{YFP}
\end{aligned} \tag{8}$$

To produce a simplified ODE model amenable to parameter inference, we start with the equations describing LuxR and LasR protein, their complexes involving C_6 and C_{12}, and the bound-/unbound promoters. Crucially, in this first derivation, we make a *rapid equilibrium* assumption for the binding reactions (5), and obtain the following relationships

$$[G_i\text{-LuxR-C}_k\text{-LuxR-C}_k]^* = K_{GDk}[G_i][\text{LuxR-C}_k\text{-LuxR-C}_k] \tag{9a}$$

$$[\text{LuxR-C}_k\text{-LuxR-C}_k]^* = K_{Dk}[\text{LuxR-C}_k]^2 \tag{9b}$$

$$[\text{LuxR-C}_k]^* = K_{Rk}[c_R][\text{C}_k] \tag{9c}$$

where $K_{Rk} = \dfrac{b_{Rk}}{\gamma + u_{Rk}}$, $K_{Dk} = \dfrac{b_{Dk}}{u_{Dk}}$ and $K_{GiDk} = \dfrac{b_{GiDk}}{u_{GiDk}}$. Therefore (also symmetry of LuxR and LasR),

$$[G_i\text{-LuxR-C}_k\text{-LuxR-C}_k]^* = K_{GiDk}K_{Dk}\left(K_{Rk}[\text{C}_k][c_R]\right)^2 \tag{10a}$$

$$[G_i\text{-LasR-C}_k\text{-LasR-C}_k]^* = K_{GiEk}K_{Ek}\left(K_{Sk}[\text{C}_k][c_S]\right)^2 \tag{10b}$$

where the new K's are defined as above.

To reduce the system to fewer variables, we consider the evolution of total LuxR, and seek to co-ordinate this with the rapid equilibrium relationships above. By denoting the total concentration of LuxR as c_R, we can write down its time-evolution as

$$\frac{d[R]_T}{dt} = \frac{d[c_R]}{dt} + \sum_k \left(\frac{d[\text{LuxR-C}_k]}{dt} + 2\frac{d[\text{LuxR-C}_k\text{-LuxR-C}_k]}{dt} + 2\sum_i \frac{d[G_i\text{-LuxR-C}_k\text{-LuxR-C}_k]}{dt} \right) \tag{11a}$$

$$= a_R - \gamma[c_R] - d_R[c_R] \dots$$

$$- \sum_k \left((\gamma + d_R^1)[\text{LuxR-C}_k] + (\gamma + d_R^2)[\text{LuxR-C}_k\text{-LuxR-C}_k] + \gamma \sum_i [G_i\text{-LuxR-C}_k\text{-LuxR-C}_k] \right) \tag{11b}$$

If there are differences between d_R, d_R^1 and d_R^2, then further reduction is complicated. However, by assuming that HSL is not protective of receiver protein ($d_R^1 = d_R^2 = d_R$), we obtain the simplification

$$\frac{dc_R}{dt} = a_R - (\gamma + d_R)c_R \tag{12}$$

Now using a conservation relationship for LuxR, we can obtain

$$c_R = [\text{LuxR}] + \sum_k \left([\text{LuxR-C}_k] + 2[\text{LuxR-C}_k\text{-LuxR-C}_k] + 2\sum_i [G_i\text{-LuxR-C}_k\text{-LuxR-C}_k] \right)$$

$$= [\text{LuxR}] + \sum_k \left(K_{Rk}[\text{LuxR}][\text{C}_k] + 2K_{Dk}K_{Rk}^2[\text{LuxR}]^2[\text{C}_k]^2 + \sum_i 2K_{GiDk}[G_i]K_{Dk}K_{Rk}^2[\text{LuxR}]^2[\text{C}_k]^2 \right)$$

When C_k is low, total LuxR is closely approximated by free LuxR, $c_R \approx [\text{LuxR}]$. But when C_k is high, c_R should be partitioned between the $[\text{LuxR-C}_k\text{-LuxR-C}_k]$ and $[G_i\text{-LuxR-C}_k\text{-LuxR-C}_k]$ species. Therefore, to simplify the analysis, we propose modelling this by using the assumption

$$c_R \approx [\text{LuxR}] + \sum_k [\text{LuxR-C}_k] = [\text{LuxR}]\left(1 + \sum_k K_{Rk}[\text{C}_k]\right) \tag{13}$$

which still captures the saturation of LuxR by C_k, using the approximations

$$[\text{LuxR}] \approx c_R . \frac{1}{1 + \sum_k K_{Rk}[\text{C}_k]} \tag{14a}$$

$$[\text{LuxR-C}_k] \approx c_R . \frac{K_{Rk}[\text{C}_k]}{1 + \sum_k K_{Rk}[\text{C}_k]} \tag{14b}$$

$$[\text{LuxR-C}_k\text{-LuxR-C}_k] \approx K_{Dk} c_R^2 \left(\frac{K_{Rk}[\text{C}_k]}{1 + \sum_k K_{Rk}[\text{C}_k]} \right)^2 \tag{14c}$$

$$[G_i\text{-LuxR-C}_k\text{-LuxR-C}_k] \approx K_{GR}^{(i)} [G_i] c_R^2 \left(\frac{K_{Rk}[\text{C}_k]}{1 + \sum_k K_{Rk}[\text{C}_k]} \right)^2 \tag{14d}$$

where $K_{GR}^{(i)} = K_{GiDk} K_{Dk}$ is assumed to be independent of which signal is bound (k), equivalent to the derivation in [3]. By symmetry, we immediately obtain equivalent expressions for interactions between LasR, HSL and PLux promoters. We denote the total concentration of LasR as c_S.

In addition to the saturation of LuxR and LasR, our reduced model also allows for saturation of G_i. By taking advantage of the conservation law

$$\begin{aligned} N_i = [G_i] + &[G_i\text{-LuxR-C}_6\text{-LuxR-C}_6] + [G_i\text{-LuxR-C}_{12}\text{-LuxR-C}_{12}] + \dots \\ &[G_i\text{-LasR-C}_6\text{-LasR-C}_6] + [G_i\text{-LasR-C}_{12}\text{-LasR-C}_{12}], \end{aligned}$$

we can derive the rate of production of mRNA as a function of c_R, c_S $[\text{C}_6]$ and $[\text{C}_{12}]$. For notational convenience we write $c_6 := [\text{C}_6]$ and $c_{12} := [\text{C}_{12}]$. Then,

$$P_i(c_6, c_{12}, c_R, c_S) = \frac{\epsilon^{(i)} + K_{GR}^{(i)} B_R + K_{GS}^{(i)} B_S}{1 + K_{GR}^{(i)} B_R + K_{GS}^{(i)} B_S} \tag{15}$$

where the fractions of bound LuxR and LasR are defined by

$$B_R := c_R^2 \left(\frac{K_{Rk} c_k}{1 + \sum_k K_{Rk} c_k} \right)^{n_R}, \qquad B_S := c_S^2 \left(\frac{K_{Sk} c_k}{1 + \sum_k K_{Sk} c_k} \right)^{n_S} \tag{16}$$

Here, we have introduce alternative exponents n_R and n_S, analogous to the usage of n in [3]. Accordingly, we obtain the following system of equations

$$\frac{d\rho}{dt} = \gamma(\rho).\rho \tag{17a}$$

$$\frac{dc_R}{dt} = a_R - (d_R + \gamma(\rho)).c_R \tag{17b}$$

$$\frac{dc_S}{dt} = a_S - (d_S + \gamma(\rho)).c_S \tag{17c}$$

$$\frac{dc_{\text{CFP}}}{dt} = a_{\text{CFP}}.P_{76}(c_6, c_{12}, c_R, c_S) - (d_{\text{CFP}} + \gamma(\rho)).c_{\text{CFP}} \tag{17d}$$

$$\frac{dc_{\text{YFP}}}{dt} = a_{\text{YFP}}.P_{81}(c_6, c_{12}, c_R, c_S) - (d_{\text{YFP}} + \gamma(\rho)).c_{\text{YFP}} \tag{17e}$$

Inference for version 1 (uniform degradation). To characterize the LuxR and LasR signalling components, we used measurements of the response of four Receiver circuits from [3] to treatment with C6 and C12 over 3-fold dilutions. The maximum LuxR and LasR production rates were normalized to the values corresponding to the Pcat promoters, as done previously [3], thus setting the scale for unobserved concentrations of LuxR and LasR.

We used (uninformative) uniform priors on the previously uncharacterized parameters, and (informative) truncated Gaussian priors on f_{480}, f_{530}, d_{CFP} and d_{YFP} with mean and standard deviation taken from the marginal posteriors of the prpr circuit characterization. We did not propagate the marginal posteriors of a_{CFP} and a_{YFP} as the promoter involved differed between the prpr circuit and Receiver circuits. The priors used are detailed in the following table.

The marginal posterior estimates are shown in Figure 19. Simulation of the maximum likelihood estimate is shown in Figure 20.

Supplementary Table 4: Priors for version 1 of the Receiver circuit characterization. In the unit column, M_R and M_S denote the normalised mass units for LuxR and LasR, and Fl denotes fluorescence units. The scaling column refers to the proposal distribution used to generate new parameters during MCMC.

Parameter	Description	Unit	Distribution	Scaling
a_{R100}	LuxR synthesis	M_R.h^{-1}	$U(1, 10^2)$	Log
a_{R33}	LuxR synthesis	M_R.h^{-1}	$U(1, 10^2)$	Log
a_{S32}	LasR synthesis	M_S.h^{-1}	$U(1, 10^2)$	Log
a_{S175}	LasR synthesis	M_S.h^{-1}	$U(1, 10^2)$	Log
d_R	LuxR degradation	h^{-1}	$U(10^{-2}, 10^2)$	Log
d_S	LasR degradation	h^{-1}	$U(10^{-2}, 10^2)$	Log
K_{R6}	Dissociation (LuxR-C6)	nM^{-1}	$U(10^{-8}, 1)$	Log
K_{S6}	Dissociation (LasR-C6)	nM^{-1}	$U(10^{-8}, 1)$	Log
K_{R12}	Dissociation (LuxR-C12)	nM^{-1}	$U(10^{-8}, 1)$	Log
K_{S12}	Dissociation (LasR-C12)	nM^{-1}	$U(10^{-8}, 1)$	Log
$K_{GR}^{(76)}$	Dissociation (P76-LuxR)	M_R^{-2}	$U(10^{-4}, 10^3)$	Log
$K_{GS}^{(76)}$	Dissociation (P76-LasR)	M_S^{-2}	$U(10^{-8}, 10^3)$	Log
$K_{GR}^{(81)}$	Dissociation (P81-LuxR)	M_R^{-2}	$U(10^{-8}, 10^3)$	Log
$K_{GS}^{(81)}$	Dissociation (P81-LasR)	M_S^{-2}	$U(10^{-4}, 10^3)$	Log
n_R	Hill constant (LuxR-HSL)	-	$U(0.5, 2)$	Real
n_S	Hill constant (LasR-HSL)	-	$U(0.5, 2)$	Real
$e^{(76)}$	Leak production (P76)	-	$U(10^{-4}, 1)$	Log
$e^{(81)}$	Leak production (P81)	-	$U(10^{-4}, 1)$	Log
a_{CFP}	CFP synthesis	Fl.cell^{-1}.h^{-1}	$U(10^2, 10^6)$	Log
a_{YFP}	YFP synthesis	Fl.cell^{-1}.h^{-1}	$U(10^2, 10^6)$	Log
b_{480}	Background fluor. (CFP)	Fl	$U(0, 10^4)$	Real
b_{530}	Background fluor. (YFP)	Fl	$U(0, 5 \times 10^3)$	Real

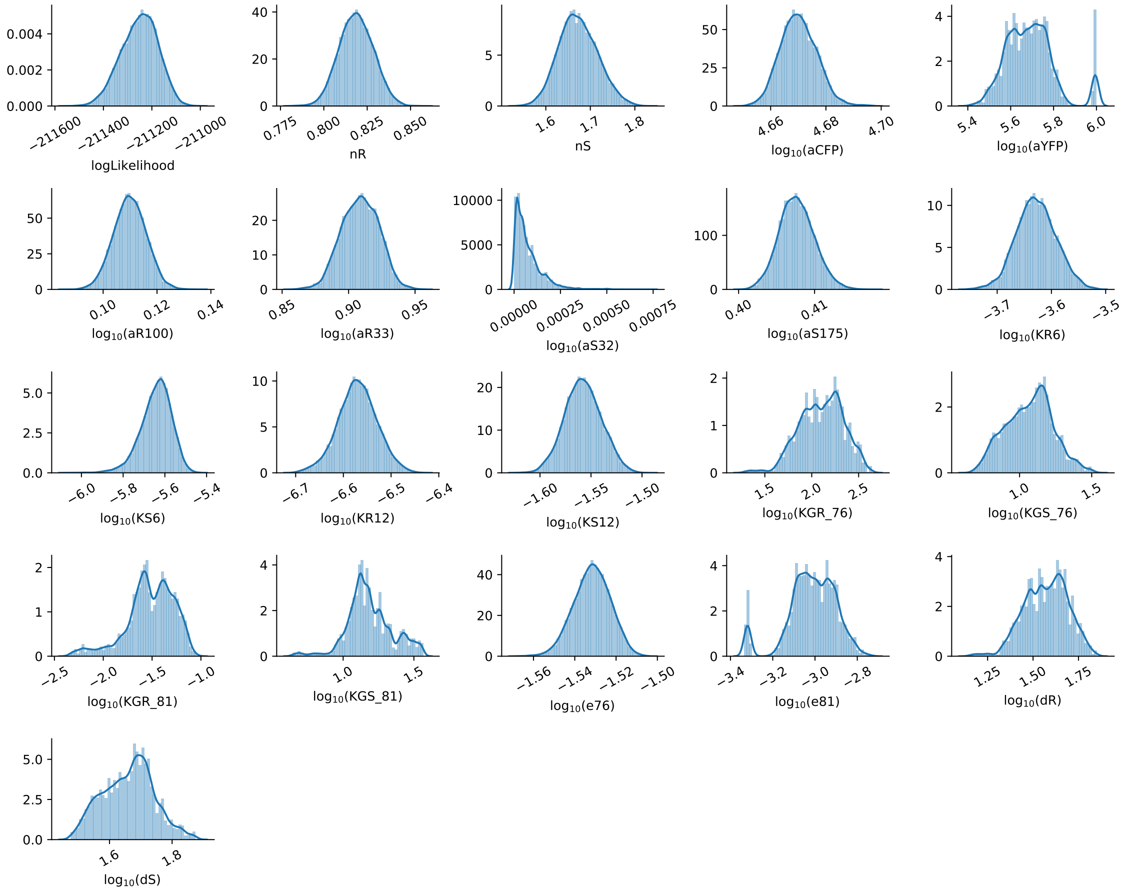

Supplementary Figure 19: Marginal parameter posterior estimates of the Receiver model (version 1) parameters. The marginal distributions are computed from 20 independent MCMC chains.

A Pcat circuit

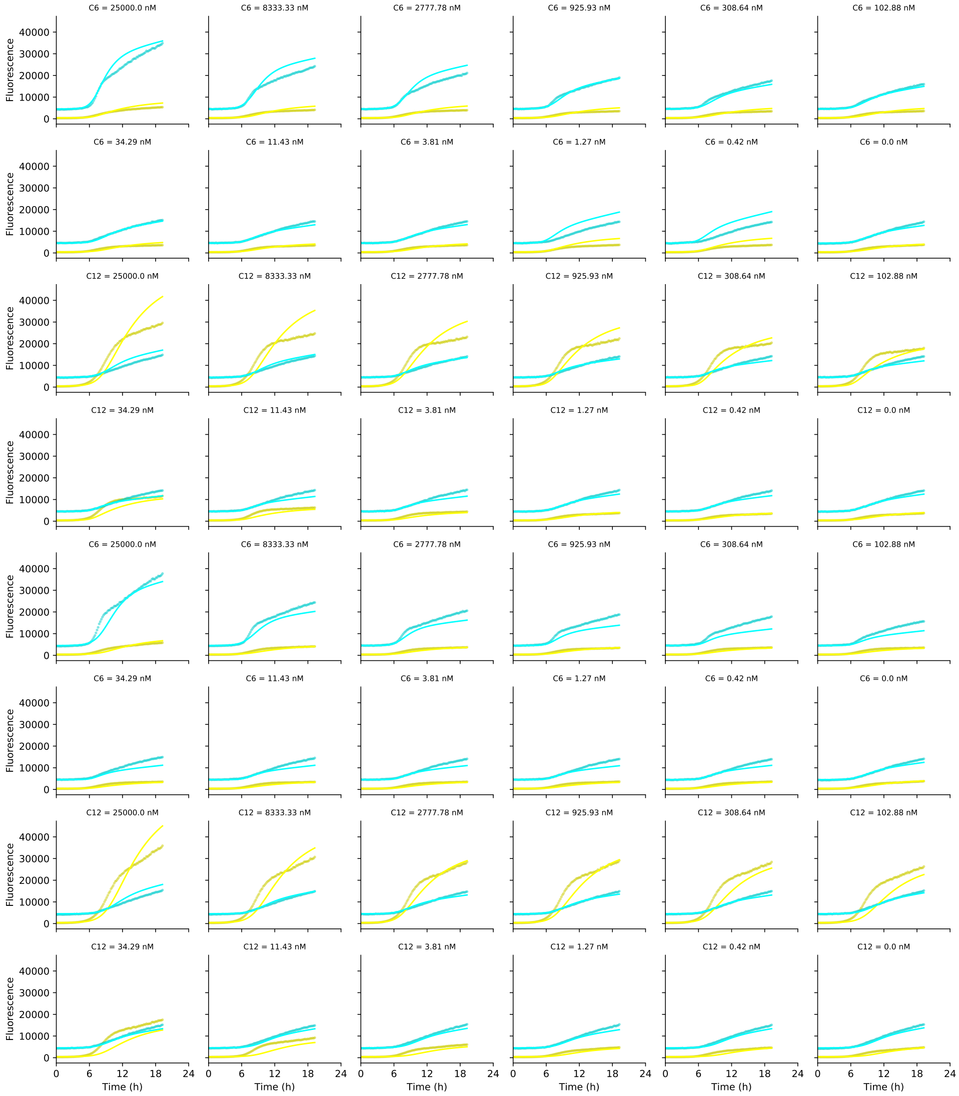

B R100S32 circuit

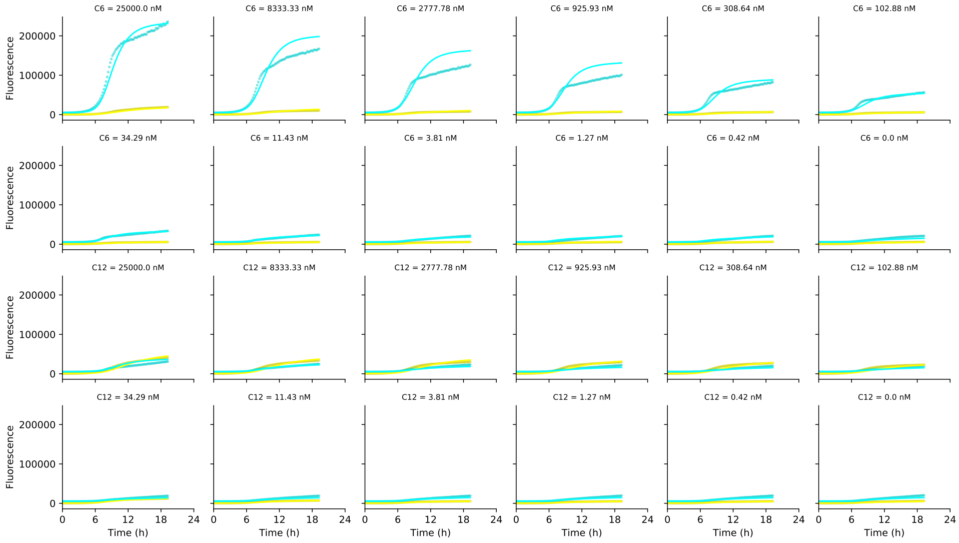

C R33S32 circuit

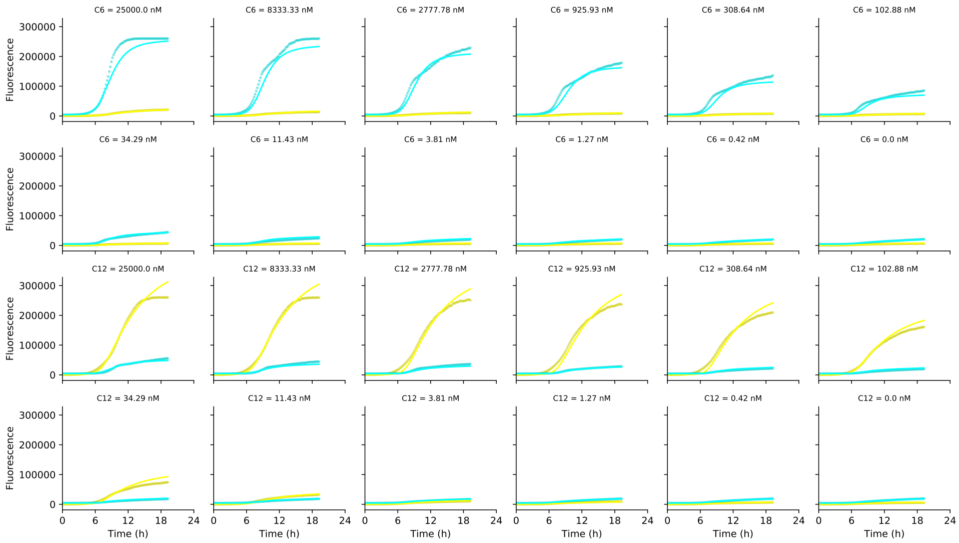

D R33S175 circuit

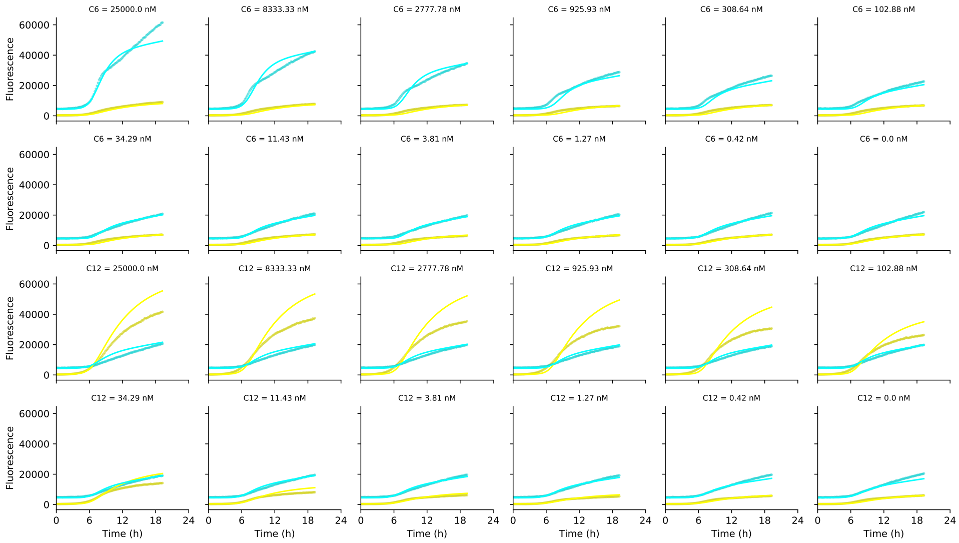

Supplementary Figure 20: Comparison of Receiver models with fluorescence measurements. Simulations are of bulk CFP (cyan lines) and YFP (yellow lines) fluorescence, evaluated with the maximum likelihood parameter set. Measurements of bulk culture fluorescence are shown in for CFP (cyan circles) and YFP (yellow circles). C6 and C12 treatment concentrations are indicated atop each panel.

Version 2 - Protected degradation. Here we provide an alternative derivation for the model reduction based on the assumption that HSL complexes and dimers are protected from degradation, i.e. that degradation predominantly occurs on the level of LuxR/LasR monomers. This is supported by in vitro analysis of purified LuxR suggesting that LuxR protein is unstable in the absence of 3OC6-HSL [6]. Furthermore, we assume that all dilution effects occur on a slow time scale as compared to the kinetic. With this derivation, we obtain the same functional structure of the promoter activities of PLux76 and PLas81 from equation (15), but with the definitions of the bound fraction of LuxR and LasR given instead by

$$B_R := c_R^2 \left(c_6^{n_R} + (e_{R12} c_{12})^{n_R} \right), \qquad B_S := c_S^2 \left((e_{S6} c_6)^{n_S} + c_{12}^{n_S} \right) \tag{18}$$

where $e_{R12} = \frac{K_{R12}}{K_{R6}}$ and $e_{S6} = \frac{K_{S6}}{K_{S12}}$ result from dividing by K_{R6} and K_{S12}. Consequently, the parameters $K_{GR}^{(i)}$ and $K_{GS}^{(i)}$ in equation (15) are rescaled by K_{R6} and K_{S12} respectively.

Inference for version 2 (Protected degradation). We carried out parameter inference for version 2 of the model directly equivalent to that done for version 1. The complete list of prior distributions for the parameters is as follows.

Supplementary Table 5: Priors for version 2 of the Receiver circuit characterization. In the unit column, M_R and M_S denote the normalised mass units for LuxR and LasR, and Fl denotes fluorescence units. The scaling column refers to the proposal distribution used to generate new parameters during MCMC.

Parameter	Description	Unit	Distribution	Scaling
a_{R100}	LuxR synthesis	$M_R.h^{-1}$	$U(1, 10^2)$	Log
a_{R33}	LuxR synthesis	$M_R.h^{-1}$	$U(1, 10^2)$	Log
a_{S32}	LasR synthesis	$M_S.h^{-1}$	$U(1, 10^2)$	Log
a_{S175}	LasR synthesis	$M_S.h^{-1}$	$U(1, 10^2)$	Log
d_R	LuxR degradation	h^{-1}	$U(10^{-2}, 10^2)$	Log
d_S	LasR degradation	h^{-1}	$U(10^{-2}, 10^2)$	Log
e_{R12}	Chemical crosstalk (LuxR)	-	$U(10^{-8}, 1)$	Log
e_{S6}	Chemical crosstalk (LasR)	-	$U(10^{-8}, 1)$	Log
$K_{GR}^{(76)}$	Dissociation (P76-LuxR)	$M_R^{-(2+n_R)}$	$U(10^{-4}, 10^3)$	Log
$K_{GS}^{(76)}$	Dissociation (P76-LasR)	$M_S^{-(2+n_S)}$	$U(10^{-8}, 10^3)$	Log
$K_{GR}^{(81)}$	Dissociation (P81-LuxR)	$M_R^{-(2+n_R)}$	$U(10^{-8}, 10^3)$	Log
$K_{GS}^{(81)}$	Dissociation (P81-LasR)	$M_S^{-(2+n_S)}$	$U(10^{-4}, 10^3)$	Log
n_R	Hill constant (LuxR-HSL)	-	$U(0.2, 2)$	Real
n_S	Hill constant (LasR-HSL)	-	$U(0.2, 2)$	Real
$e^{(76)}$	Leak production (P76)	-	$U(10^{-4}, 1)$	Log
$e^{(81)}$	Leak production (P81)	-	$U(10^{-4}, 1)$	Log
a_{CFP}	CFP synthesis	$Fl.cell^{-1}.h^{-1}$	$U(10^2, 10^6)$	Log
a_{YFP}	YFP synthesis	$Fl.cell^{-1}.h^{-1}$	$U(10^2, 10^6)$	Log
B_{480}	Background fluorescence (CFP)	Fl	$U(0, 10^4)$	Real
B_{530}	Background fluorescence (YFP)	Fl	$U(0, 5 \times 10^3)$	Real

The marginal posterior estimates are shown in Figure 21. Simulation of the maximum likelihood estimate is shown in Figure 22.

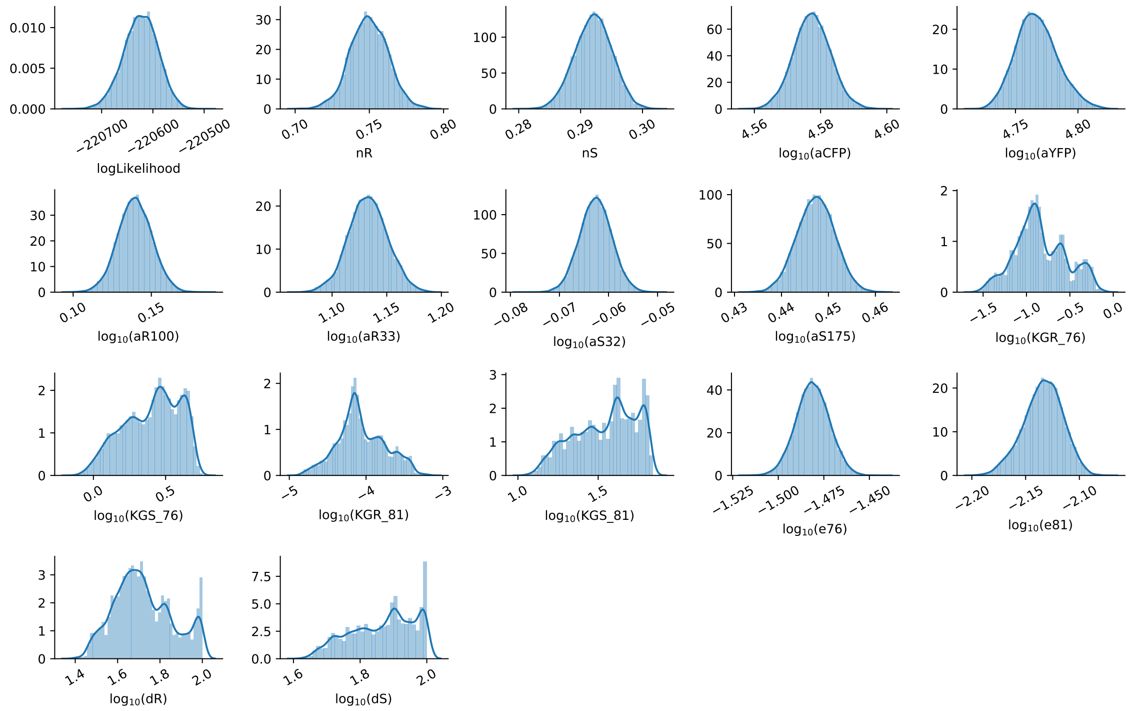

Supplementary Figure 21: Marginal parameter posterior estimates of the Receiver model (version 2) parameters. The marginal distributions are computed from 20 independent MCMC chains.

A Pcat circuit

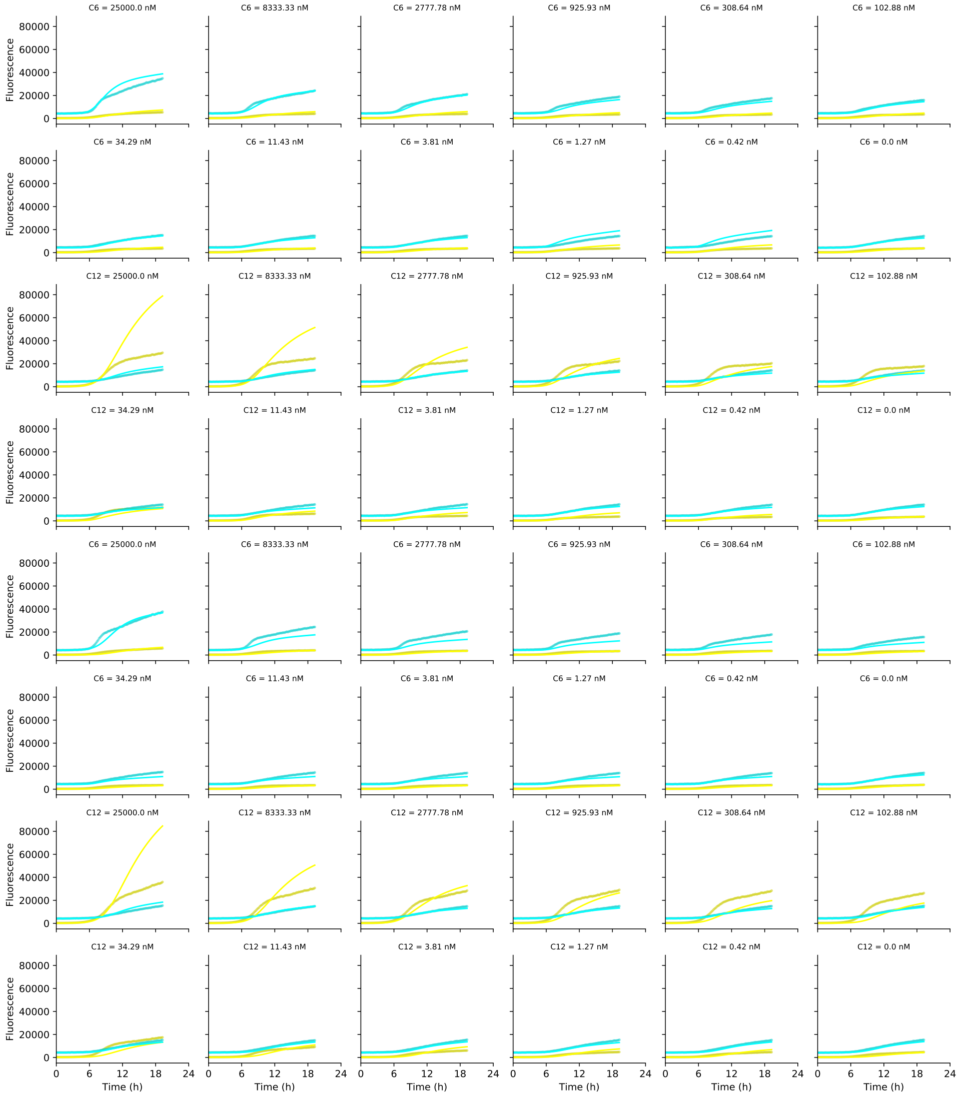

B R100S32 circuit

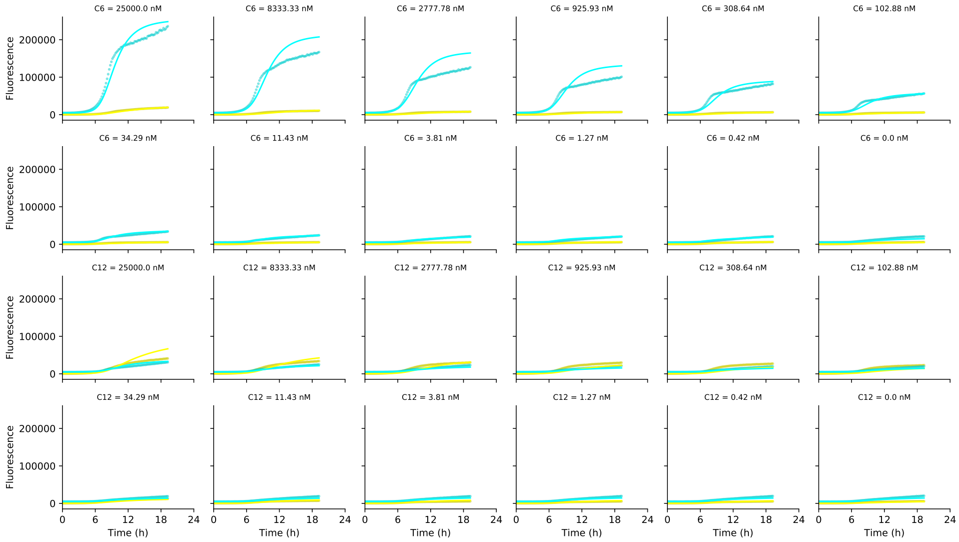

C R33S32 circuit

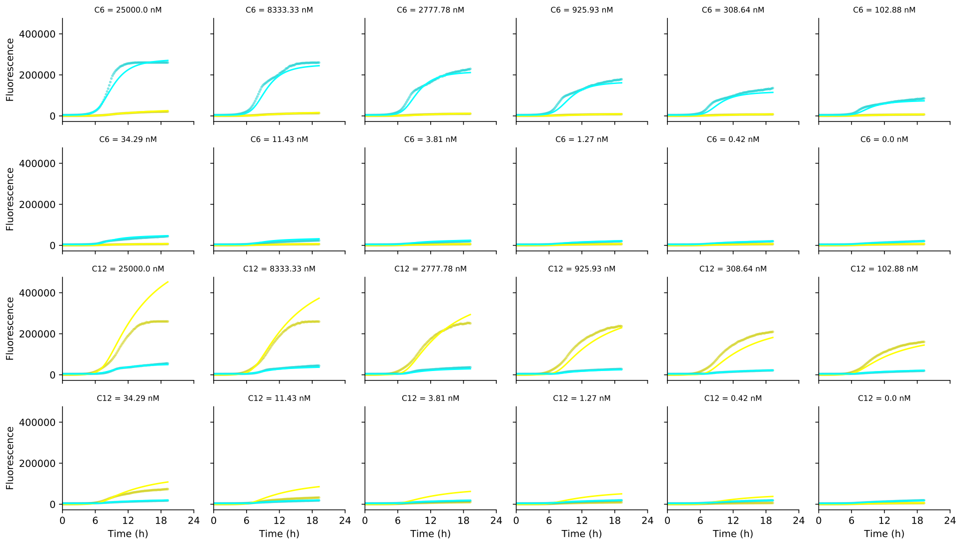

D R33S175 circuit

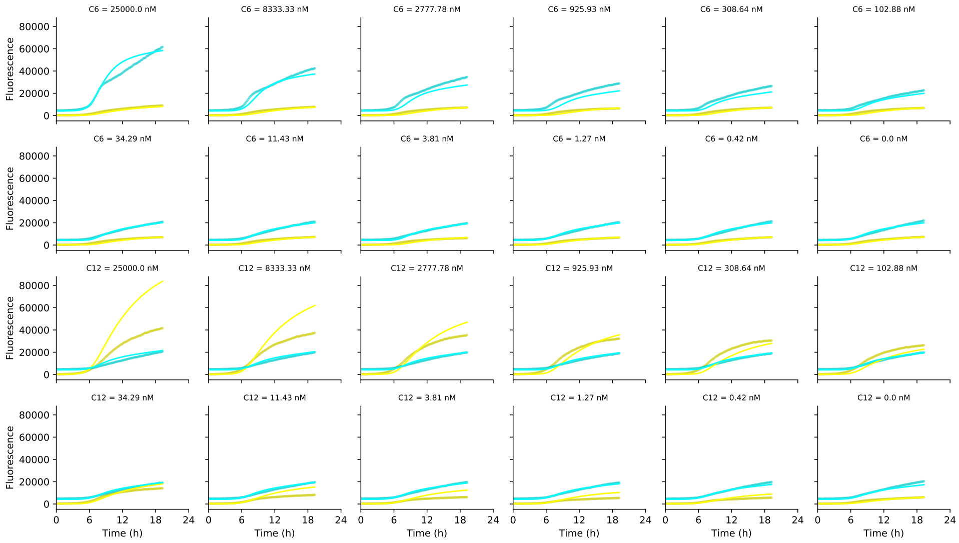

Supplementary Figure 22: Comparison of Receiver models with fluorescence measurements. Simulations are of bulk CFP (cyan lines) and YFP (yellow lines) fluorescence, evaluated with the maximum likelihood parameter set. Measurements of bulk culture fluorescence are shown in for CFP (cyan circles) and YFP (yellow circles). C6 and C12 treatment concentrations are indicated atop each panel.

Exclusive Receiver model

To model the Exclusive Receiver, we consider the inhibition of PTet by TetR and PLac by LacI, and the mechanism of chemical inhibition by IPTG and ATC. For promoter regulation, we use the inhibition Hill function

$$H_I(x, n) := \frac{1}{1 + x^n} \tag{19}$$

Typically, these functions would include a parameter for the half-saturation concentration, but we omit that here because, without loss of generality, [LacI] and [TetR] can be arbitrarily scaled by those half-saturation concentrations. In such a rescaling, the half-saturation constants get embedded within the maximal production rates, a_L and a_T. For the chemical inhibitors, we assume a reaction of the form

$$\text{inhibitor} + \text{repressor} \to \text{sequestered product} \tag{20}$$

Correspondingly, the action of IPTG and ATC is proportional to the product of its concentration and its target repressor protein concentration.

$$\frac{d\rho}{dt} = \gamma(\rho).\rho \tag{21a}$$

$$\frac{dc_{\text{YFP}}}{dt} = a_{\text{YFP}}.f_{81}(c_6, c_{12}, c_R, c_S) - (\gamma(\rho) + d_{\text{YFP}})c_{\text{YFP}} \tag{21b}$$

$$\frac{dc_{\text{CFP}}}{dt} = a_{\text{CFP}}.f_{76}(c_6, c_{12}, c_R, c_S) - (\gamma(\rho) + d_{\text{CFP}})c_{\text{CFP}} \tag{21c}$$

$$\frac{dc_{530}}{dt} = b_Y - \gamma(\rho).c_{530} \tag{21d}$$

$$\frac{dc_{480}}{dt} = b_C - \gamma(\rho).c_{480} \tag{21e}$$

$$\frac{dc_R}{dt} = a_R.H_I(c_T, n_T) - (\gamma(\rho) + d_R).c_R \tag{21f}$$

$$\frac{dc_S}{dt} = a_S.H_I(c_L, n_L) - (\gamma(\rho) + d_S).c_S \tag{21g}$$

$$\frac{dc_L}{dt} = a_L.P_{76}(c_6, c_{12}, c_R, c_S) - (\gamma(\rho) + d_L + i_I.c_I).c_L \tag{21h}$$

$$\frac{dc_T}{dt} = a_T.P_{81}(c_6, c_{12}, c_R, c_S) - (\gamma(\rho) + d_T + i_A.c_A).c_T \tag{21i}$$

where P_{76} and P_{81} are defined in (15).

Inference for version 1 (uniform degradation). The inference procedure was less robust for the Exclusive Receiver, as compared with upstream circuits in the inference graph. In particular, we found it was not possible to identify a unique mode within the parameter space when all parameters were allowed to be flexible. Specifically, there was strong interdependency within the triplet $\{a_L, d_L, i_I\}$ and the triplet $\{a_T, d_T, i_A\}$. Our interpretation is that we are unable to fully recover the time-scales of variations in c_L and c_T, as they are likely to not vary much during the experiments we used for characterization. It's likely that c_L and c_T quickly stabilise to equilibria when the cells are transferred to the media containing the treatments (specific concentrations of C6, C12, IPTG and ATC). In which case, only those equilibrium values will be identifiable, and not the production and degradation rates separately. Therefore, in the final version of the inference results presented here, we have fixed the degradation rates of LacI and TetR to 1 h^{-1}.

Even when running the inference with d_L and d_T fixed, we found that chain convergence was not perfect, and some chains got stuck in local optima. Therefore, in our marginal posterior estimates, we have only included chains that converged to relatively good likelihood scores Figure 23. The marginals clearly indicate some additional flexibility in the inferred parameter values, possibly resulting from the larger parameter space being navigated, which includes uninformative priors for the parameters listed in Table 6, but also some flexibility in all of the parameters associated with the Receiver module, despite them having a strong prior.

Supplementary Table 6: Priors for the Exclusive Receiver circuit characterization. In the unit column, M_L and M_T denote the normalised mass units for LacI and TetR, and Fl denotes fluorescence units. The scaling column refers to the proposal distribution used to generate new parameters during MCMC.

Parameter	Description	Unit	Distribution	Scaling
a_L	LacI synthesis	$M_L.\text{h}^{-1}$	$U(1, 10^4)$	Log
a_T	TetR synthesis	$M_T.\text{h}^{-1}$	$U(1, 10^4)$	Log
d_L	LacI degradation	h^{-1}	1 (Fixed)	N/A
d_T	TetR degradation	h^{-1}	1 (Fixed)	N/A
n_L	Hill constant (LacI)	-	$U(0.5, 4.0)$	Real
n_T	Hill constant (TetR)	-	$U(0.5, 4.0)$	Real
i_A	ATC inhibition of TetR	$M_T^{-1}.(\text{ng/ml ATC})^{-1}.\text{h}^{-1}$	$U(10^{-2}, 10^3)$	Log
i_I	IPTG inhibition of LacI	$M_L^{-1}.(\text{mM IPTG})^{-1}.\text{h}^{-1}$	$U(10^{-2}, 10^3)$	Log
B_{480}	Background fluor. (CFP)	Fl	$U(0, 10^4)$	Real
B_{530}	Background fluor. (YFP)	Fl	$U(0, 5 \times 10^3)$	Real

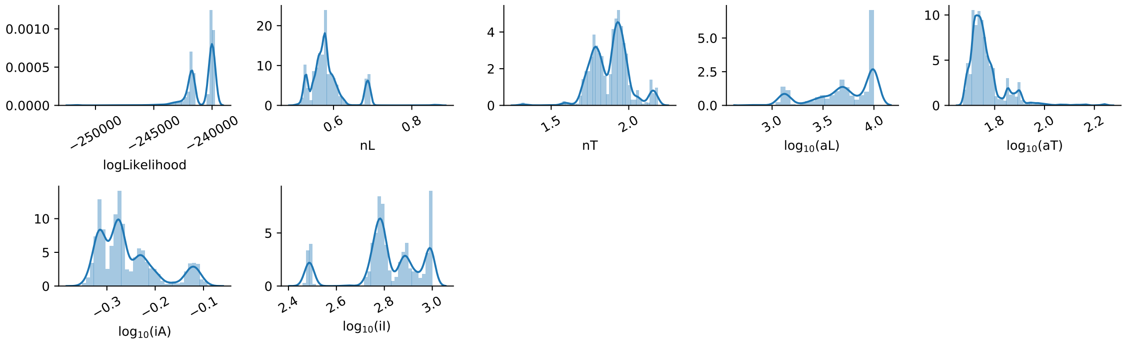

Supplementary Figure 23: Marginal parameter posterior estimates of the Exclusive Receiver model (version 1) parameters. The marginal distributions are computed from 9 of 20 independent MCMC chains. Chains were discarded that did not converge to high log-likelihood regions.

Simulation of the maximum likelihood estimate is shown in Figure 23.

A 2d dilution of C_6 and C_{12}

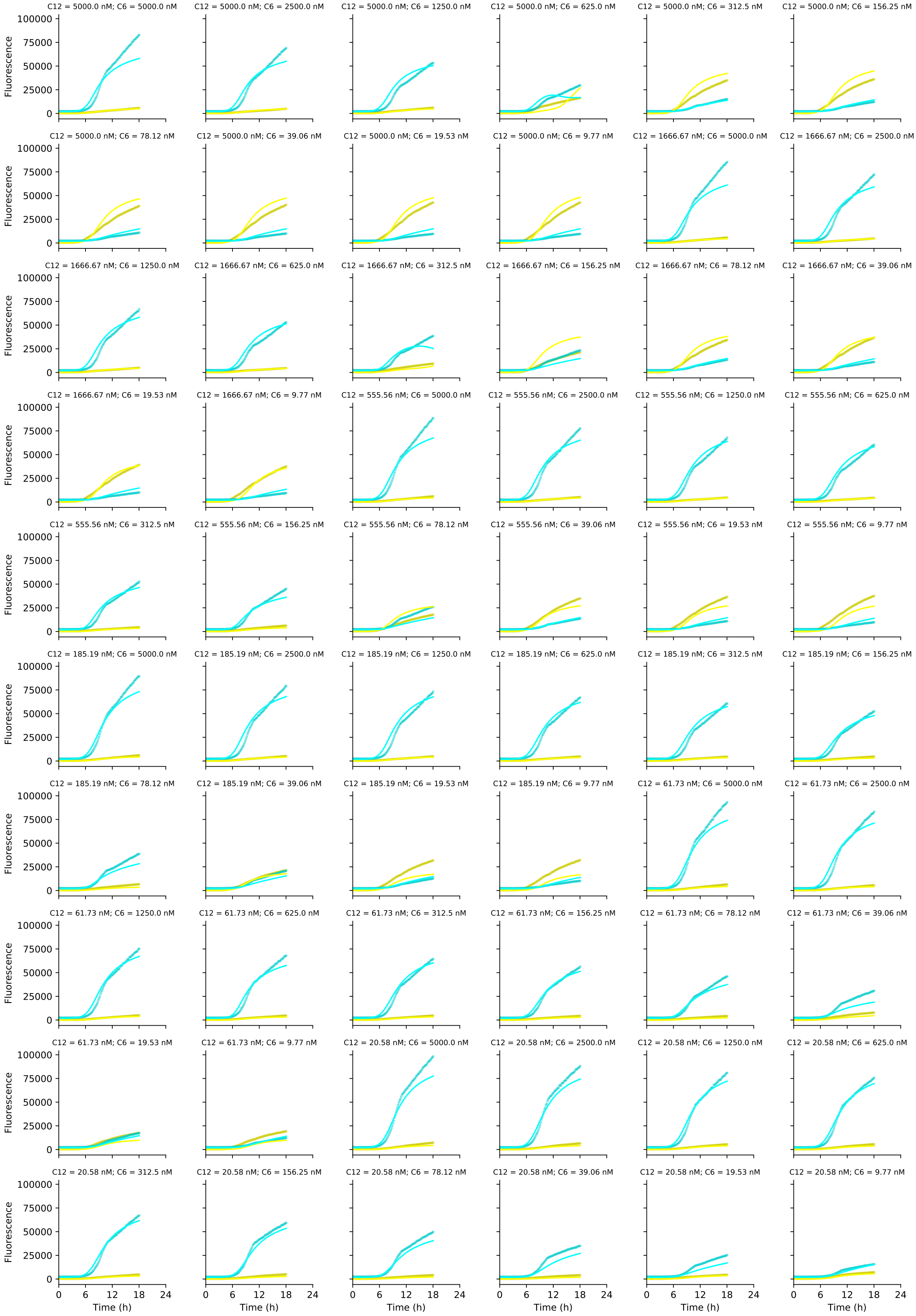

B Characterization of IPTG and ATC

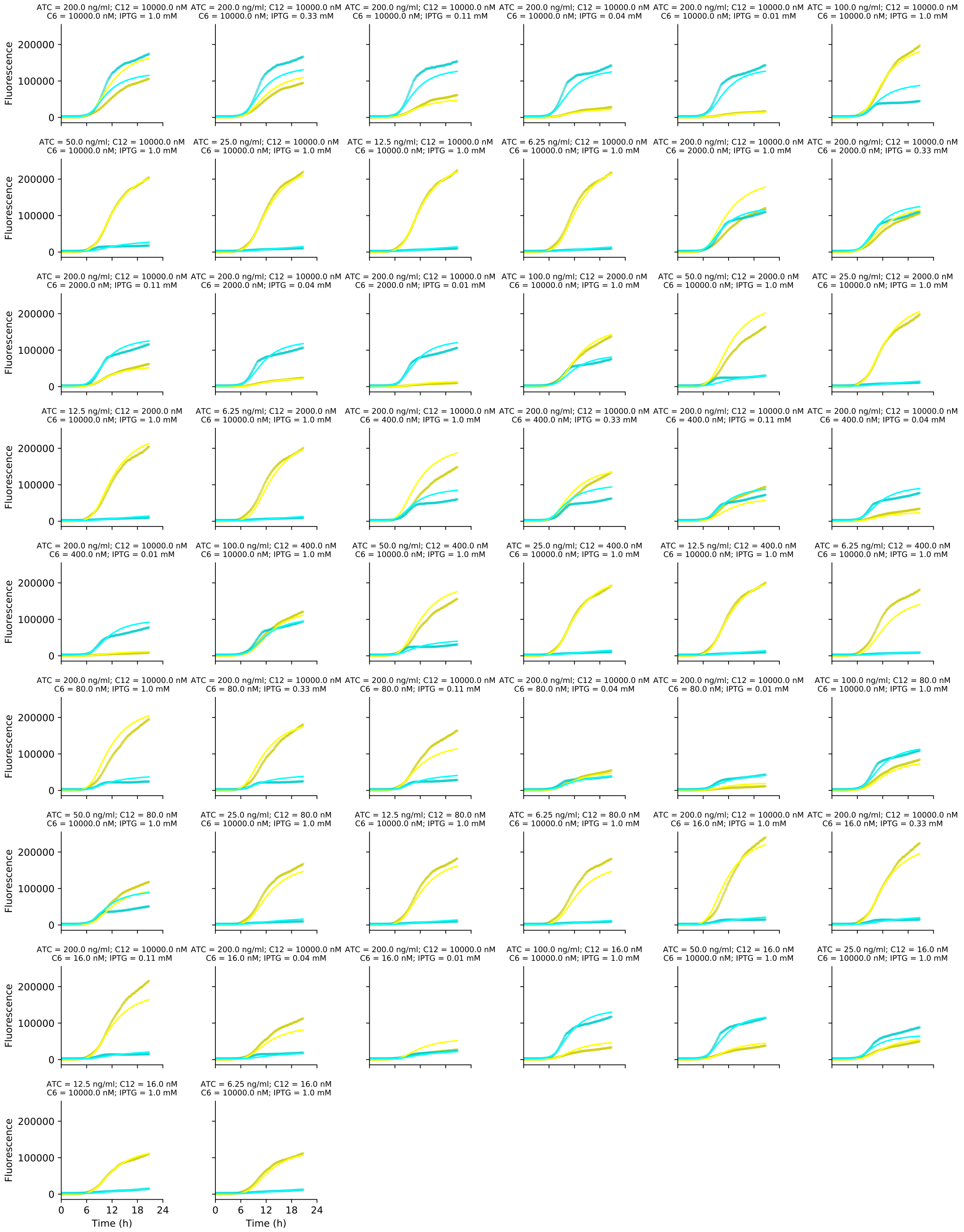

Supplementary Figure 23: Comparison of Exclusive Receiver model (version 1) with fluorescence measurements. Simulations are of bulk CFP (cyan lines) and YFP (yellow lines) fluorescence, evaluated with the maximum likelihood parameter set. Measurements of bulk culture fluorescence are shown in for CFP (cyan circles) and YFP (yellow circles). C6, C12, ATC and IPTG treatment concentrations are indicated atop each panel.

Inference for version 2 (Protected degradation). The inference procedure was also not completely robust for version 2 of the Exclusive Receiver model. We used the same uninformative priors for the parameters specific to the Exclusive Receiver model as in version 1, including fixing d_L and d_T. Again, in our marginal posterior estimates, we have only included chains that converged to relatively good likelihood scores Figure 24.

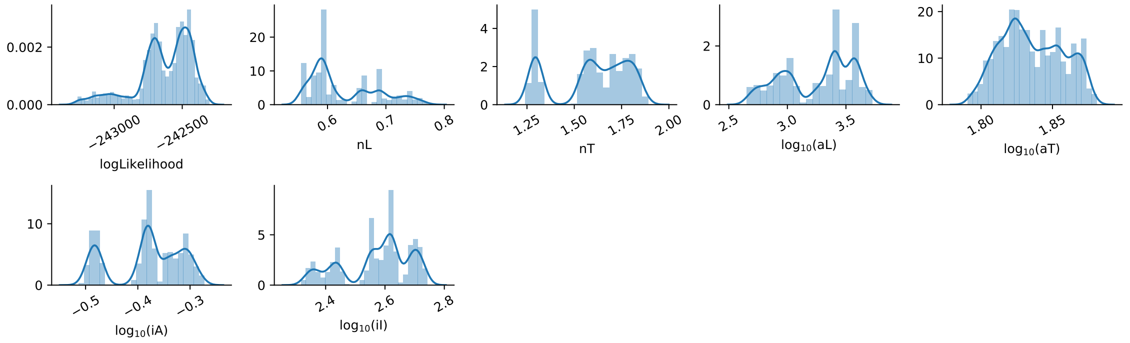

Supplementary Figure 24: Marginal parameter posterior estimates of the Exclusive Receiver model (version 2) parameters. The marginal distributions are computed from 8 of 20 independent MCMC chains. Chains were discarded that did not converge to high log-likelihood regions.

Simulation of the maximum likelihood estimate is shown in Extended Data Figure 2.

2.2 Bistability Analysis

In this section we outline computations used to create Figure 2b in the main text which compares regions of bistability indicated by hysterisis experiments in flow cytometry to that given by the differential equation model for the exclusive receiver circuit.

To characterize the region in the (c_{12}, c_6) plane where bistability occurs, we used numerical continuation to calculate a co-dimension two limit curve. The code in `get_bifurcations.jl` in our repository takes advantage of the Julia package `PseudoArcLengthContinuation.jl` [7]. To calculate the bifurcations, we only need consider the steady states of the model. The auto-fluorescence equations are independent of the others and CFP and YFP are simply readouts of c_L and c_T respectively, leaving only four coupled equations to solve, defined by state vector $\mathbf{c} = (c_R, c_S, c_L, c_T)$. For simplicity of analysis, we assume that cell density ρ is constant, and consequently the specific growth rate $\gamma(\rho) =: \gamma_0$ is constant. Later, we check this assumption in Figure 26. With this simplification, the model given by (21) can be represented compactly as

$$\frac{d\mathbf{c}}{dt} = \mathbf{F}_\theta(\mathbf{c}, \mathbf{u}) \tag{22}$$

where $\mathbf{u} = (c_6, c_{12}, c_I, c_A)$ are the experimental control parameters, θ is the vector containing the inferred parameters from Section S1 and γ_0.

To improve the numerical stability of numerical continuation, we transform the model into $\log_{10}$ coordinates via the element-wise transformations $\mathbf{c} \to 10^{\mathbf{c}}$ and $\mathbf{u} \to 10^{\mathbf{u}}$ yielding

$$\frac{d\mathbf{c}}{dt} = \frac{\mathbf{F}_\theta(10^{\mathbf{c}}, 10^{\mathbf{u}})\, 10^{-\mathbf{c}}}{\ln(10)} \tag{23}$$

The steady states are defined by zeros of the numerator of the right-hand side. We can immediately see that this transformation induced a zero at $\mathbf{c} \to \infty$ which we are not interested in and thus simply seek to solve

$$\mathbf{F}_\theta(10^{\mathbf{c}}, 10^{\mathbf{u}}) = 0 \tag{24}$$

To further increase numerical stability of finding 24 we explicitly calculate the Jacobian in the log coordinate system. Luckily the Jacobian in the new coordinates can be expressed in terms of the matrix product between the Jacobian in the original coordinates and the Jacobian of the coordinate transformation

$$\frac{\partial \mathbf{F}_\theta}{\partial \mathbf{c}} \to \ln(10) \left.\frac{\partial \mathbf{F}_\theta}{\partial \mathbf{c}}\right|_{\mathbf{c}\to 10^{\mathbf{c}}} \mathrm{Diag}[\,10^{\mathbf{c}}] \tag{25}$$

where $\mathrm{Diag}[\mathbf{v}]$ is a diagonal matrix with the vector components of $\mathbf{v}$ along the diagonal. The Jacobian in the original coordinates is

$$\frac{\partial \mathbf{F}_\theta}{\partial \mathbf{c}} = \begin{pmatrix} 0 & 0 & 0 & a_{R33}\frac{\partial H_T}{\partial T} \\ 0 & 0 & a_{S175}\frac{\partial H_L}{\partial L} & 0 \\ a_L\frac{\partial P_{76}}{\partial R} & a_L\frac{\partial P_{76}}{\partial S} & 0 & 0 \\ a_T\frac{\partial P_{81}}{\partial R} & a_T\frac{\partial P_{81}}{\partial S} & 0 & 0 \end{pmatrix} - \mathrm{Diag}\left[\gamma_0 + \begin{pmatrix} d_R \\ d_S \\ d_L \\ d_T \end{pmatrix} + \begin{pmatrix} 0 \\ 0 \\ i_I c_I \\ i_A c_A \end{pmatrix}\right] \tag{26}$$

where partials of inhibitory hill functions and promoter activities are

$$\frac{\partial H_X}{\partial X} = \frac{-n_X.c_X^{(n_X-1)}}{(1+c_X^{n_X})^2} \qquad \frac{\partial P_N}{\partial X} = \frac{K_{GX}^N\,(1-e_N)}{\left[1+K_{GR}^N B_R(c_R) + K_{GS}^N B_S(c_S)\right]^2}\frac{\partial B_X}{\partial X} \tag{27}$$

and partial bound molecules for uniform and protected degradation models are respectively

$$\frac{\partial B_X}{\partial X} = 2c_X\frac{(K_{X6}c_6)^{n_X} + (K_{X12}c_{12})^{n_X}}{(1+K_{X6}c_6 + K_{X12}c_{12})^{n_X}} \qquad \frac{\partial B_X}{\partial X} = 2c_X\begin{cases} c_6^{n_X} + (E_{R12}c_{12})^{n_X} & X = R \\ c_{12}^{n_X} + (E_{S6}c_6)^{n_X} & X = S \end{cases}$$

Given the rate function $\mathbf{F}_\theta$ and its Jacobian $\frac{\partial \mathbf{F}_\theta}{\partial \mathbf{c}}$ in log coordinates we can perform a co-dimension one parameter continuation for a fixed value of c_{12} along the c_6 direction to find a limit point. Then the solution can be continued along a limit curve in the (c_6, c_{12}) plane along both directions until the limits of the observation region are met. Figure 25 reveals these curves for different models and inferred maximum likelihood parameter sets θ and Figure 26 reveals that the chosen model for the main text is insensitive to changes in growth γ_0 and therefore we can safely assume that the qualitative behaviour of the model will not change if the cell density is constant. For simplicity, this is what is done in the spatial simulations in Sections 2.3 and 2.4.

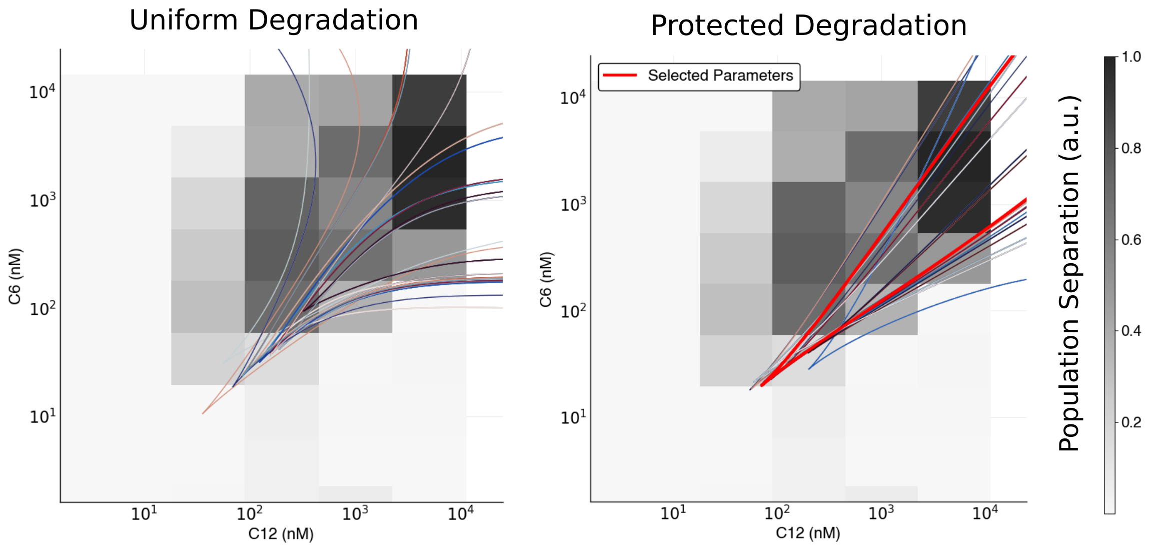

Supplementary Figure 25: Bifurcation curves for uniform and protected degradation models. Curves are plotted on top of bimodal population separation heatmaps generated from flow cytometry data. The selected maximum likelihood parameters are used in the main text.

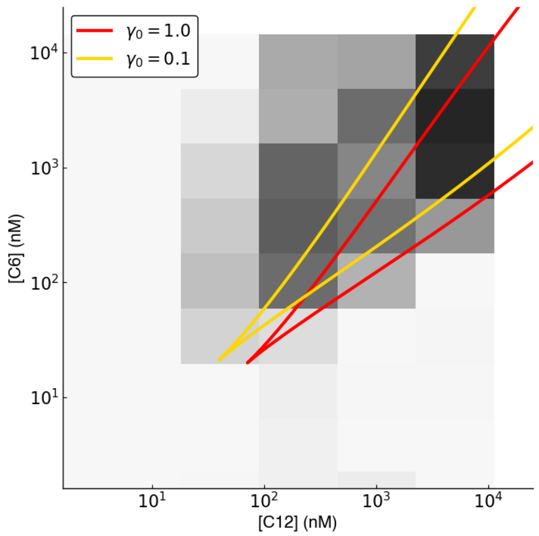

Supplementary Figure 26: Bifurcation curve insensitivity specific growth rate γ_0. The cell density is a monotonically increasing function of time that saturates at the carrying capacity. Concomitantly, the specific growth rate declines towards 0. By evaluating the bifurcation diagram at different values of γ_0, we can see how the cusp would move over time. Here the maximum likelihood parameters from the main text reveal that the cusp does not move much as the cell density increases over time.

2.3 Boundary Experiments

Simulation and local equilibria

For spatial simulations a simple forward-Euler method is implemented in `get_movie.py`. The bacterial colonies for the spatial experiments were placed on top of agar with no signalling molecules inside it. Then additional volumes of agar were attached either side of the width of the experiment with different concentrations of c_6 and c_{12}. The signalling molecules then diffuse in the agar and established a cross-gradient felt by the bacterial colonies. These are governed by diffusion

$$\frac{\partial c_X(x,t)}{\partial t} = D_X \frac{\partial^2 c_X(x,t)}{\partial x^2} \tag{28}$$

where $D_6 = 1.8 \cdot 10^{-6}$ m^2.h^{-1} and $D_{12} = 0.9 \cdot 10^{-6}$ m^2.h^{-1}.

The initial conditions for spatial simulations are zero everywhere except for $c_6(x,t)|_{t=0}$ and $c_{12}(x,t)|_{t=0}$ initialised in small regions widths w on opposite sides of the experiment of width W at concentrations such that the homogeneous equilibrium after diffusion would be $c_6(x,t)|_{t\to\infty} = \overline{C6}$ and $c_{12}(x,t)|_{t\to\infty} = \overline{C12}$ with zero-flux boundary conditions. Therefore

$$c_6(x,t)|_{t=0} = \frac{W\overline{C6}}{w} H(w-x) \qquad c_{12}(x,t)|_{t=0} = \frac{W\overline{C12}}{w} H(x-W+w) \tag{29}$$

where $H(x)$ is a unit step function.

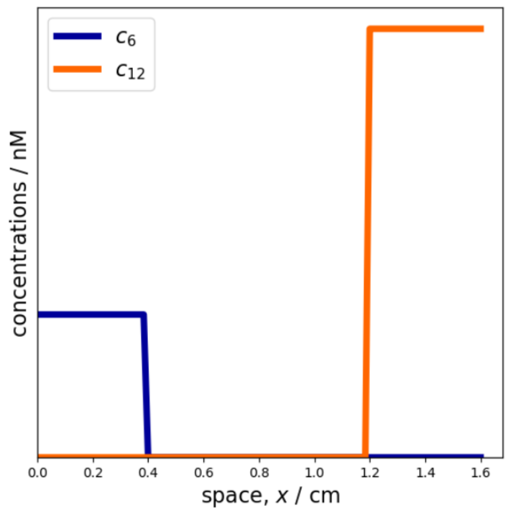

Supplementary Figure 27: Initial conditions $c_6(x,t)|_{t=0}$ and $c_{12}(x,t)|_{t=0}$ with $W = 1.6$ cm and $w = 0.4$ cm

Each location x experiences concentrations c_6, c_{12} which define a local equilibrium for the remaining state variables. These local equilibria evolve over time and are chased by the actual concentrations of protein in the cells at that location. As shown by Figure 25, at some concentrations c_6, c_{12} there may two stable equilibria rather than one. Therefore it becomes useful to not only display the dynamics in the one dimensional spatial domain but also in the (c_6, c_{12}) plane. How and when local equilibria bifurcate reveals the eventual fate of the spatial pattern.

Figure 28 shows a snapshot of the dynamics in the spatial domain x and the (c_6, c_{12}) plane. A sharp boundary in space forms when the state density in (c_6, c_{12}) moves into the bistable region enclosed by the limit point curve. This means that, given the cross-gradient initial conditions 29, if the homogeneous equilibrium $\overline{C6}, \overline{C12}$ lies within the bistable region, a sharp stationary boundary will form. If $\overline{C6}, \overline{C12}$ lies below the cusp of the limit point curve, only soft boundaries will form. In other regions outside the bistable cone the sharp boundary has a finite velocity and will eventually leave the experimental region. See supplementary movies 2-5 for examples of the above. This motivated the experimental exploration of the space of $\overline{C6}, \overline{C12}$ and measurements of boundary velocity, which are described in the following section.

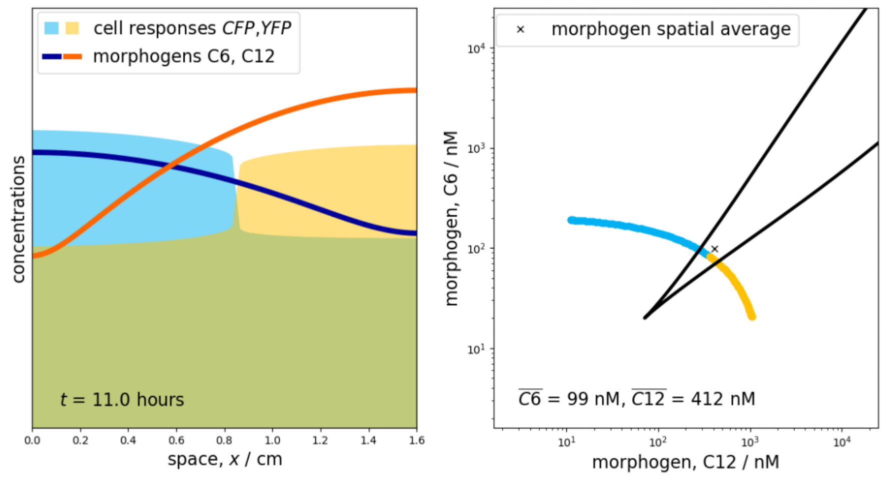

Supplementary Figure 28: Dynamics in the spatial domain x on the left and (c_6, c_{12}) plane on the right. Spatial averages of morphogens $\overline{C6}, \overline{C12}$ are 99 nM and 412 nM respectively for both panels

Computation of the boundary velocity

The velocity of the boundary is determined using `get_movement.py` from the TIFF image stack $M[t, x, y, s]$ obtained by the fluorescence microscope from one experiment, set up with a chosen $\overline{C6}, \overline{C12}$ combination. Here t indexes the time point, x and y index the width and height and s indexes the three channels: CFP,YFP and RFP.

First the data are normalised by the RFP channel. This way the location of the boundary can be defined by comparing the pixel values of one channel against the other. The pixels are masked for the colony grid squares $(x, y) \in \Omega$ which are otherwise surrounded by hydrophobic ink filter paper, on which no colonies grow. The grid squares are detected by thresholding the constituent RFP channel at the end time point.

$$X[t, x, y, s] := \frac{M[t, x, y, s]}{M[t, x, y, \bullet]} \quad \text{where} \quad (x, y) \in \Omega \tag{30}$$

$$\text{where} \quad \Omega := \left\{ (x, y) : M[\mathbf{end}, x, y, \bullet] > \frac{1}{2} \right\} \tag{31}$$

Next, a sigmoidal basis function Bayesian Ridge regressor is applied to each channel and time point along the width x of the preprocessed data $X[t, x, y, s]$. Sigmoidal basis functions are used because they satisfy the zero-flux boundary conditions as well as our expectations that the fluorescence profile across the width of the experiment will mostly be flat with a transient step due to the cross-gradient. The unknown weights ϕ of the basis functions are obtained by minimising the objective

$$J_\phi[t, s] := \sum_{(x,y)\in\Omega} ||X[t, x, y, s] - f_\phi(x)[t, s]||^2 \tag{32}$$

$$\text{where} \quad f_\phi(x) := \sum_{(\alpha,\mu,\sigma)\in\phi} \frac{\alpha}{1 + e^{-\frac{x-\mu}{\sigma}}} \tag{33}$$

With the continuous estimate $f_{\phi^*}(x)$ for each $[t, s]$, the location of the boundary can be obtained even if it was estimated to lie between two colonies as seen in Figure 29. Since this continuous estimate is obtained for each point in time, the position of the boundary can be tracked in a smooth kymograph as shown in Figure 30. We define the boundary location $\beta[t]$ to be where the estimate of the CFP channel • is equal to that of the YFP channel •,

$$f_{\phi^*}(\beta)[t, \textcolor{orange}{\bullet}] = f_{\phi^*}(\beta)[t, \textcolor{cyan}{\bullet}] \tag{34}$$

The distance travelled by the boundary $\Delta\beta$ from its formation time t^* to the end as a fraction of the size of the experiment W can now be computed. The formation time t^* was judged by eye and seems to lie between $3-5$ h, at which fluorescence values are sufficiently steep to form a sharp boundary. The boundary should have travelled at least 10% along the width - which is the approximate size of one colony grid square - in order to be classified as moving.

$$\Delta\beta = \frac{\beta[t^*] - \beta[end]}{W} \tag{35}$$

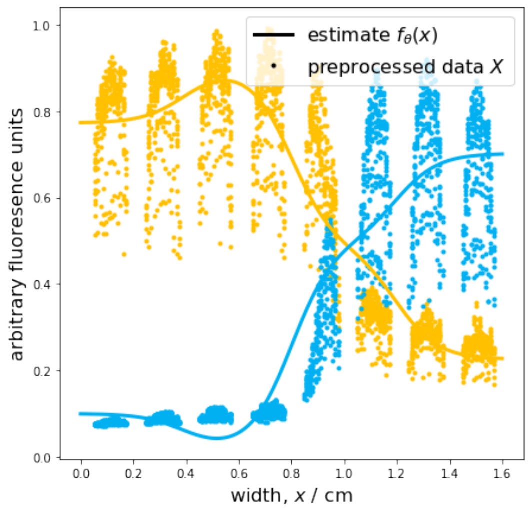

Supplementary Figure 29: Bayesian Ridge regression estimate $f_\phi(x)$ from preprocessed data X

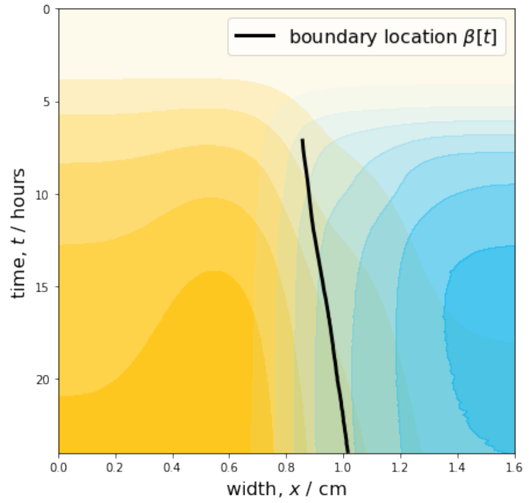

Supplementary Figure 30: Boundary location $\beta[t]$ by equating estimates from two channels

The distance travelled $\Delta\beta$ can be investigated for different equilibrium concentrations $\overline{C6}$ and $\overline{C12}$. Figure 31 shows results for a two dimensional dilution between 5 nM and 25000 nM and fixed 10 μM IPTG. The subsequent classification using $\Delta\beta$ is shown as Figure 3c in the main text.

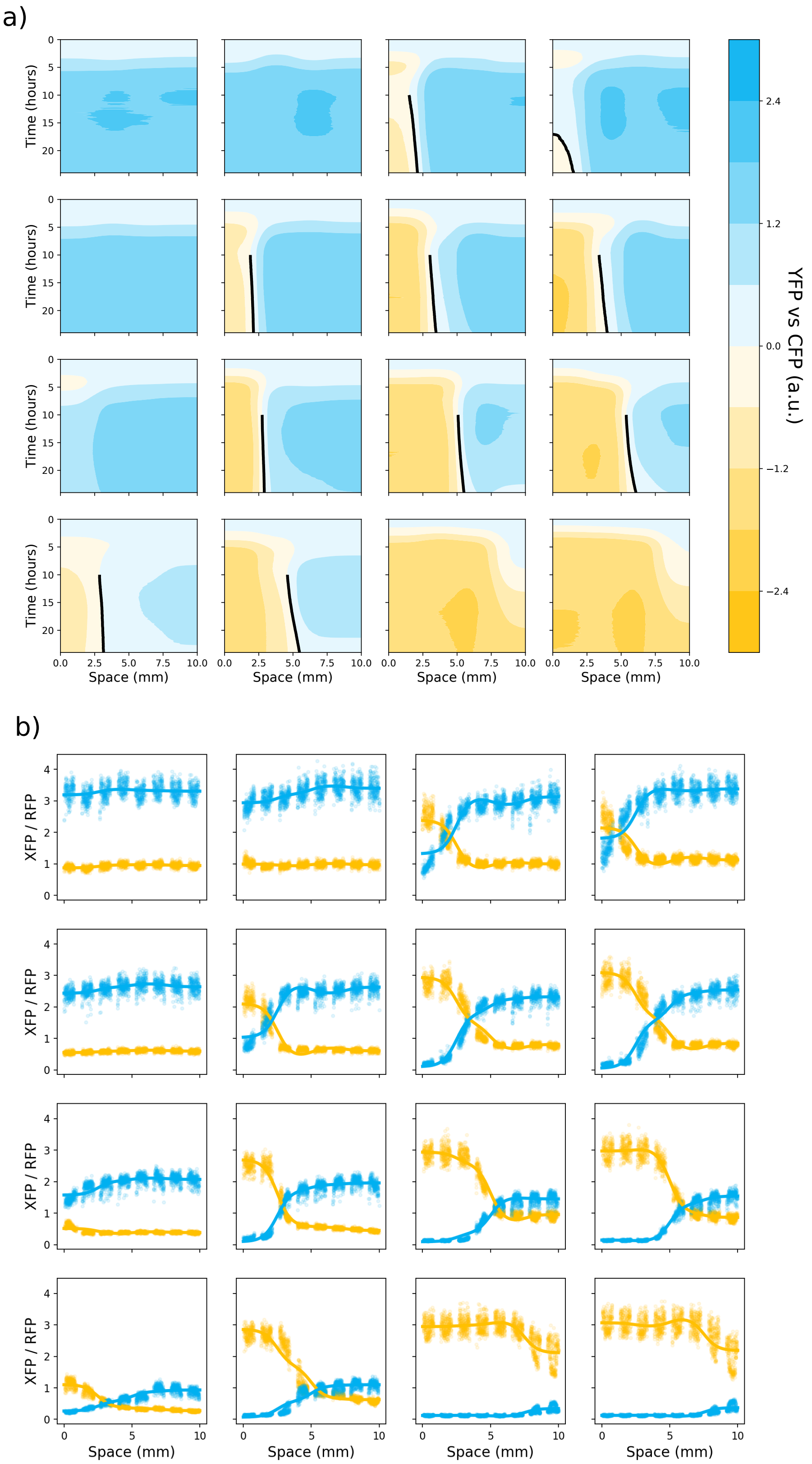

Supplementary Figure 31: a) Boundary movements $\beta[t]$ for a grid of equilibrium concentrations $\overline{C6}, \overline{C12}$ and fixed 10 μM IPTG. This is an expanded version of Figure 3c in the main text, showing the kymograpghs that were used for classification. b) Bayesian Ridge regression estimates for final time points of preprocessed data X

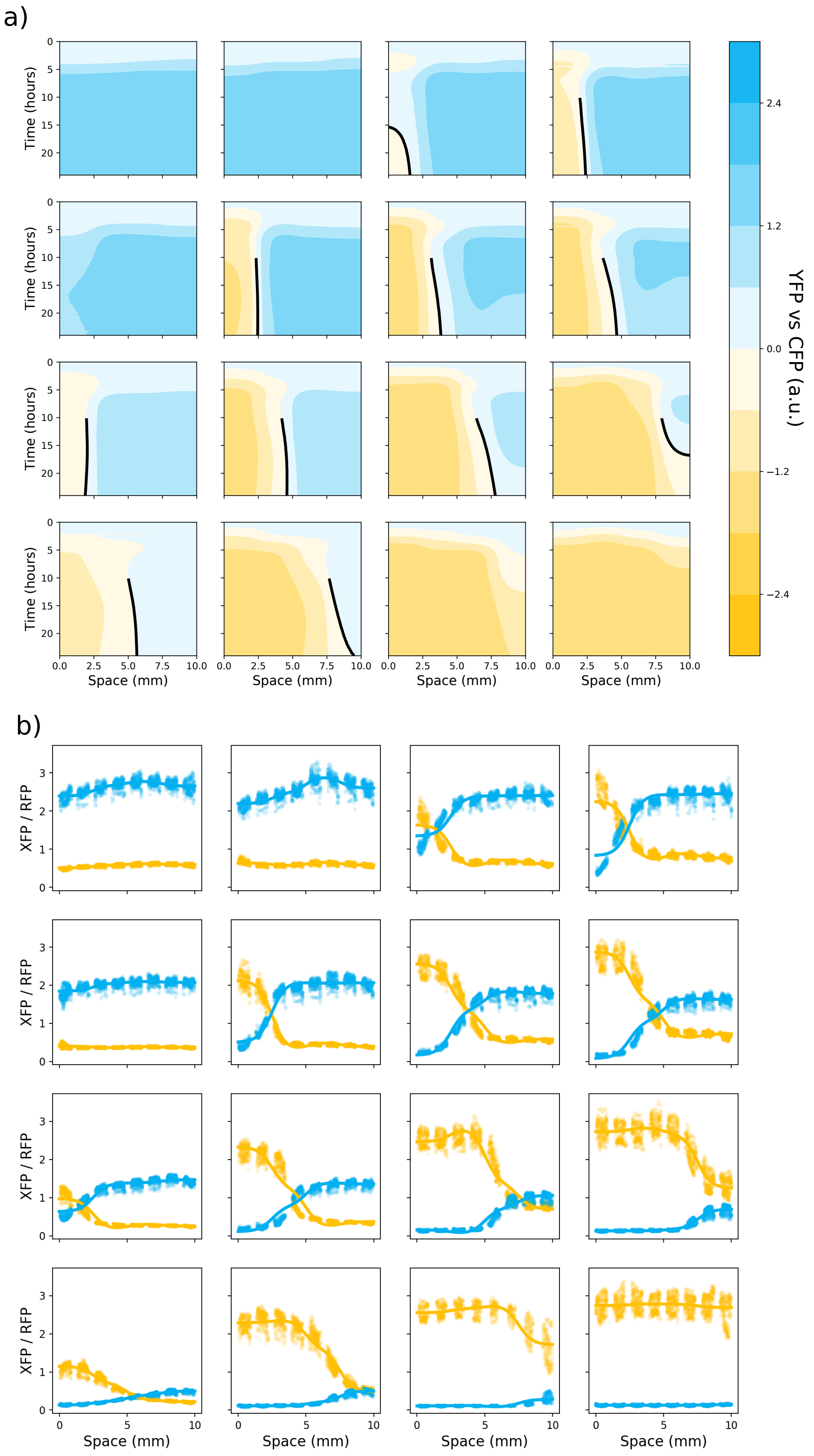

Supplementary Figure 32: a) Replicate of Figure 31 for a two dimensional dilution of $\overline{C6}, \overline{C12}$ between 5 nM and 25000 nM and fixed 10 μM IPTG. b) Bayesian Ridge estimates for final time points of preprocessed data X

a)

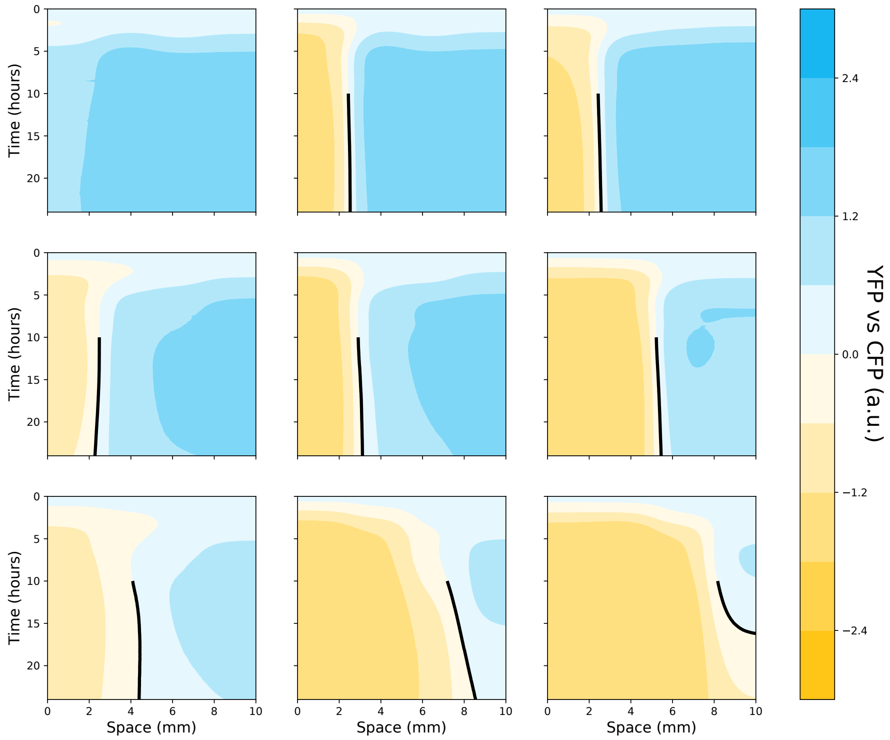

b)

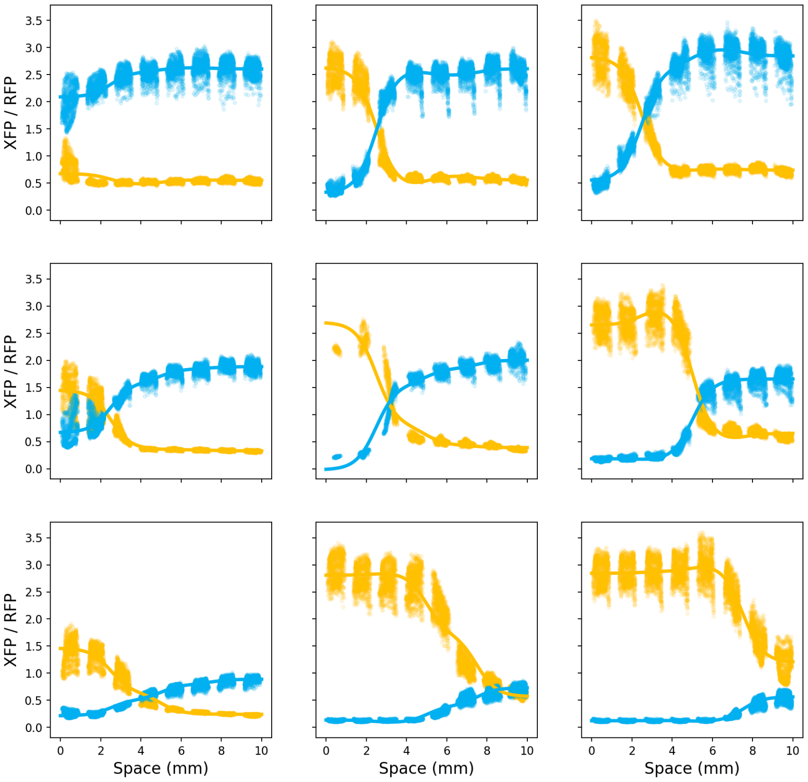

Supplementary Figure 33: a) Replicate of Figure 31 for a two dimensional dilution of $\overline{C6}, \overline{C12}$ between 20 nM and 2000 nM and fixed 10 μM IPTG. b) Bayesian Ridge estimates for final time points of preprocessed data X

Use of IPTG to influence bifurcation curve

Solid culture experiments on boundary movement were initially performed in the absence of IPTG. We observed stationary boundaries at the concentrations labelled as grey points (Figure S24). The shape of the region encompassing the grey points was qualitatively similar to the conical bistability region we observed in previous experiments and in our models, but was quantitatively shifted such that even very low concentrations of C6 enabled bistability. We hypothesized that this was due to minor differences in culture conditions between solid and liquid cultures. We hypothesized that the addition of a low concentration of IPTG would partially derepress LacI, thereby making the YFP-dominant region larger and more like that seen in liquid culture. We used IPTG to shift the bistability region back (Figure S25) to coincide with the region in liquid culture. This allowed us to perform the solid culture experiments in a regime in which we could observe the transition from stable boundary to moving boundary with YFP dominance, by using higher concentrations of C12 while keeping C6 constant.

Experiments with the relay circuit (Fig. 4b) performed as expected without the need for addition of IPTG or ATC.

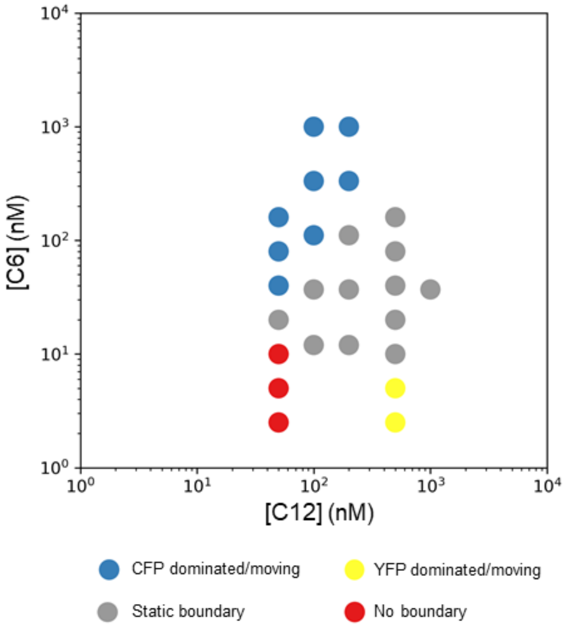

Supplementary Figure 34: Boundary movement in the absence of IPTG Boundaries were classified by eye at the spatial average concentrations indicated.

Experiments with the relay circuit (4a) performed as expected without the addition of IPTG or ATC.

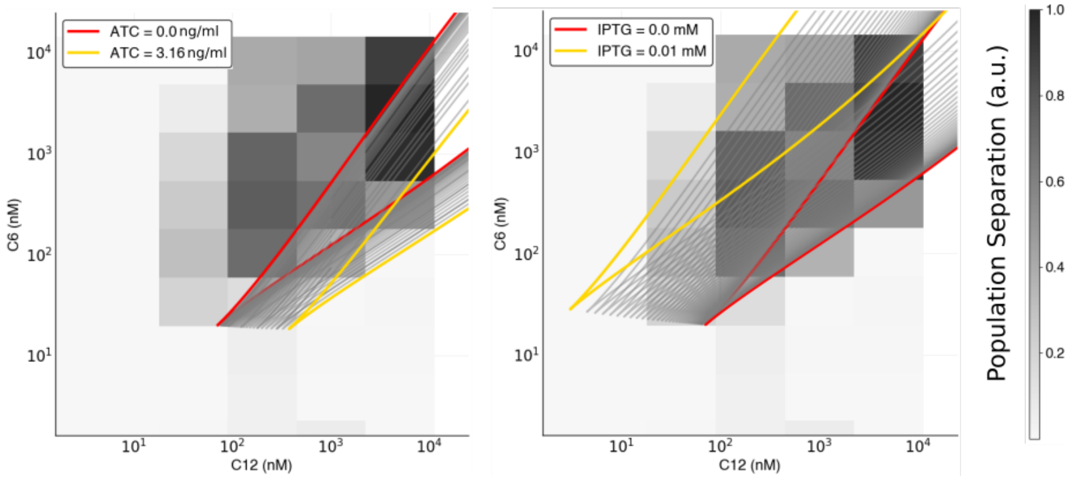

Supplementary Figure 35: Bifurcation curve dependence on ATC and IPTG. Increasing the concentrations c_A or c_I shifts the cusp point keeping the general shape of the bistable region. Here the maximum likelihood parameters from the main text are used as the reference cusp.

2.4 Models of the Exclusive Receiver Relay Circuits

The Exclusive Receiver Relay circuits were described in Figure 4 of the main text. The models for these circuits are simple extensions of the constant density model of the Exclusive Receiver circuit (22) with $\rho = \rho_0$, $\gamma = \gamma_0$ and production terms in the signal diffusion equations

$$\frac{\partial c_6(x,t)}{\partial t} = D_6 \frac{\partial^2 c_6(x,t)}{\partial x^2} + k_{C6}\rho_0 c_{\text{luxI}} \qquad \frac{\partial c_{12}(x,t)}{\partial t} = D_{12} \frac{\partial^2 c_{12}(x,t)}{\partial x^2} + k_{C12}\rho_0 c_{\text{lasI}} \tag{36}$$

$$\frac{dc_{\text{luxI}}}{dt} = P_{81} - (\gamma_0 + d_{\text{luxI}})c_{\text{luxI}} \qquad \frac{dc_{\text{lasI}}}{dt} = P_{76} - (\gamma_0 + d_{\text{lasI}})c_{\text{lasI}} \tag{37}$$

Here, the additional production terms $k_{C6}\rho_0 c_{\text{luxI}}$ and $k_{C12}\rho_0 c_{\text{lasI}}$ break mass conservation of the signalling molecules and thus may increase the spatial averages $\overline{C6}, \overline{C12}$. These terms can be visualised as vertical and horizontal vector fields components in the (c_6, c_{12}) plane respectively, which induce a drift on the local equilibria and the spatial average $\overline{C6}, \overline{C12}$. Since there is no saturation of signal production, the spatial average $\overline{C6}, \overline{C12}$ will eventually always move outside of the bistable region, giving rise to a uniform dominant CFP or YFP profile. Within the finite observation time $t < 24$ h however, we may observe sharp boundaries forming, as the spatial average $\overline{C6}, \overline{C12}$ passes through the bistable region. Figure 36 shows how even monotonic gradients can facilitate boundary formation.

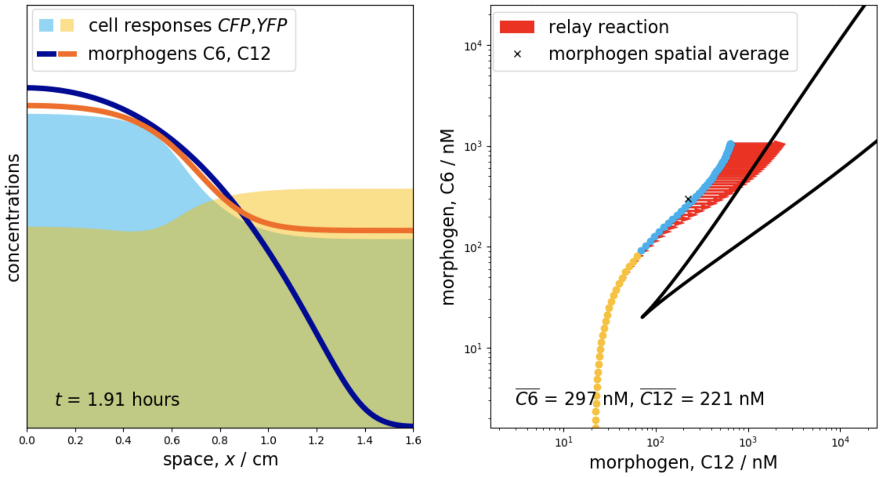

Supplementary Figure 36: Dynamics of the relay circuit for $k_{C12} > 0, k_{C6} = 0$ in the spatial domain x on the left and (c_6, c_{12}) plane on the right, showing additional reaction terms as red vectors driving the dynamics. This way even monotonic gradients can facilitate boundary formation

The additional parameters k_{C6},k_{C12},d_{luxI},d_{lasI} are tuned by hand. Simulations in Section 2.3 suggest that local cellular responses approach local equilibria faster than the diffusion timescales of signalling molecules. This means we can reasonably assume that our system is diffusion-limited and so reactive dynamics (37) are much faster than diffusive dynamics (36) and we can apply the quasi-steady state assumption to (36) yielding

$$\frac{\partial c_6(x,t)}{\partial t} = D_6 \frac{\partial^2 c_6(x,t)}{\partial x^2} + \frac{k_{C6}\rho_0 P_{81}}{\gamma_0 + d_{\text{luxI}}}, \qquad \frac{\partial c_{12}(x,t)}{\partial t} = D_{12} \frac{\partial^2 c_{12}(x,t)}{\partial x^2} + \frac{k_{C12}\rho_0 P_{76}}{\gamma_0 + d_{\text{lasI}}}, \tag{38}$$

and therefore in effect only two ratio parameter ratios need to be tuned. These ratios determine the magnitude of the relay reactions in the (c_6, c_{12}) plane, which determine whether diffusion had contracted the local equilibria to the spatial average $\overline{C6}, \overline{C12}$ before or after having crossed into bistable region. Boundaries only form if contraction to $\overline{C6}, \overline{C12}$ occurred after entering the bistable region. See supplementary movies 6-7 for examples of these cases.

References

[1] Boyan Yordanov, Neil Dalchau, Paul K Grant, Michael Pedersen, Stephen Emmott, Jim Haseloff, and Andrew Phillips. A computational method for automated characterization of genetic components. *ACS synthetic biology*, 3(8):578–588, 2014.

[2] Timothy J. Rudge, James R. Brown, Fernan Federici, Neil Dalchau, Andrew Phillips, James W. Ajioka, and Jim Haseloff. Characterization of Intrinsic Properties of Promoters. *ACS Synthetic Biology*, 5(1):89–98, jan 2016.

[3] Paul K Grant, Neil Dalchau, James R Brown, Fernan Federici, Timothy J Rudge, Boyan Yordanov, Om Patange, Andrew Phillips, and Jim Haseloff. Orthogonal intercellular signaling for programmed spatial behavior. *Molecular Systems Biology*, 12(1):849, 2016.

[4] Neil Dalchau, Paul K. Grant, Prashant Vaidyanathan, Carlo Spaccasassi, Colin Gravill, and Andrew Phillips. Scalable dynamic characterization of synthetic gene circuits. *bioRxiv*, page 635672, aug 2019.

[5] Geoffrey Roeder, Paul K Grant, Andrew Phillips, Neil Dalchau, and Edwards Meeds. Efficient amortised bayesian inference for hierarchical and nonlinear dynamical systems. In *International Conference on Machine Learning (ICML 2019)*, 2019.

[6] ML Urbanowski, CP Lostroh, and EP Greenberg. Reversible acyl-homoserine lactone binding to purified vibrio fischeri luxr protein. *Journal of Bacteriology*, 186(3):631–637, 2004.

[7] Romain Veltz. PseudoArcLengthContinuation.jl, March 2019.
