# Supplementary material for: Interpretation of morphogen gradients by a synthetic bistable circuit
Source: Nat Commun. 2020 Nov 2;11:5545. doi: 10.1038/s41467-020-19098-w (PMC7608687; doi:10.1038/s41467-020-19098-w)
Supplement: Supplementary file 2 — Reporting Summary [file 41467_2020_19098_MOESM2_ESM.pdf]

nature research

Corresponding author(s): Paul K. Grant and Andrew Phillips

Last updated by author(s): Sep 15, 2020

Reporting Summary

Nature Research wishes to improve the reproducibility of the work that we publish. This form provides structure for consistency and transparency in reporting. For further information on Nature Research policies, see our Editorial Policies and the Editorial Policy Checklist.

Statistics

For all statistical analyses, confirm that the following items are present in the figure legend, table legend, main text, or Methods section.

n/a	Confirmed	
☒	☐	The exact sample size (*n*) for each experimental group/condition, given as a discrete number and unit of measurement
☒	☐	A statement on whether measurements were taken from distinct samples or whether the same sample was measured repeatedly
☒	☐	The statistical test(s) used AND whether they are one- or two-sided<br>*Only common tests should be described solely by name; describe more complex techniques in the Methods section.*
☒	☐	A description of all covariates tested
☒	☐	A description of any assumptions or corrections, such as tests of normality and adjustment for multiple comparisons
☐	☒	A full description of the statistical parameters including central tendency (e.g. means) or other basic estimates (e.g. regression coefficient) AND variation (e.g. standard deviation) or associated estimates of uncertainty (e.g. confidence intervals)
☒	☐	For null hypothesis testing, the test statistic (e.g. *F*, *t*, *r*) with confidence intervals, effect sizes, degrees of freedom and *P* value noted<br>*Give P values as exact values whenever suitable.*
☐	☒	For Bayesian analysis, information on the choice of priors and Markov chain Monte Carlo settings
☒	☐	For hierarchical and complex designs, identification of the appropriate level for tests and full reporting of outcomes
☒	☐	Estimates of effect sizes (e.g. Cohen's *d*, Pearson's *r*), indicating how they were calculated

Our web collection on statistics for biologists contains articles on many of the points above.

Software and code

Policy information about availability of computer code

Data collection	BMG FluoStar Omega Reader Control Software 5.10R2; FACSDiva 8.01; LASX 3.6.0.20104; MetaMorph software (version 7.8.10.0, Molecular Devices, USA); MATLAB 2014a
Data analysis	FCS Express 7; Schnitzcells (release 1.1, 2005); Custom code will be made available in a GitHub repository at https://github.com/gszep/double-exclusivereporter

For manuscripts utilizing custom algorithms or software that are central to the research but not yet described in published literature, software must be made available to editors and reviewers. We strongly encourage code deposition in a community repository (e.g. GitHub). See the Nature Research guidelines for submitting code & software for further information.

Data

Policy information about availability of data

All manuscripts must include a data availability statement. This statement should provide the following information, where applicable:

- Accession codes, unique identifiers, or web links for publicly available datasets
- A list of figures that have associated raw data
- A description of any restrictions on data availability

Source data are provided with this paper. Plate fluorometer and flow cytometry datasets can be found in supplementary file sourcedata.zip. Raw microscopy images (figures 2c, 3a, 4b) are available on request to the authors. The exclusiver receiver plasmid is available from Addgene (Addgene ID 160376).

Field-specific reporting

Please select the one below that is the best fit for your research. If you are not sure, read the appropriate sections before making your selection.

- [x] Life sciences
- [ ] Behavioural & social sciences
- [ ] Ecological, evolutionary & environmental sciences

For a reference copy of the document with all sections, see nature.com/documents/nr-reporting-summary-flat.pdf

Life sciences study design

All studies must disclose on these points even when the disclosure is negative.

Sample size	No sample size calculation was performed. 3-4 biologicial replicates were performed based on standard practices and previous experience with these experimentation techniques.
Data exclusions	No data was excluded.
Replication	3-4 biological replicates were performed for most experiments. In all experiments performed, genetic circuits functioned in a manner consistent with the analysis and conclusions presented in this study.
Randomization	There is no reason to believe that location on plate would affect results, therefore experiments were not randomized.
Blinding	Data analysis was performed using code such as Schnitzells and custom code found at https://github.com/gszep/double-exclusivereporter so individual investigator preferences were irrelevant and blinding was not required.

Reporting for specific materials, systems and methods

We require information from authors about some types of materials, experimental systems and methods used in many studies. Here, indicate whether each material, system or method listed is relevant to your study. If you are not sure if a list item applies to your research, read the appropriate section before selecting a response.

Materials & experimental systems

n/a	Involved in the study	
[x]	[ ]	Antibodies
[x]	[ ]	Eukaryotic cell lines
[x]	[ ]	Palaeontology and archaeology
[x]	[ ]	Animals and other organisms
[x]	[ ]	Human research participants
[x]	[ ]	Clinical data
[x]	[ ]	Dual use research of concern

Methods

n/a	Involved in the study	
[x]	[ ]	ChIP-seq
[ ]	[x]	Flow cytometry
[x]	[ ]	MRI-based neuroimaging

Flow Cytometry

Plots

Confirm that:

- [x] The axis labels state the marker and fluorochrome used (e.g. CD4-FITC).
- [x] The axis scales are clearly visible. Include numbers along axes only for bottom left plot of group (a 'group' is an analysis of identical markers).
- [x] All plots are contour plots with outliers or pseudocolor plots.
- [x] A numerical value for number of cells or percentage (with statistics) is provided.

Methodology

Sample preparation	After experimental treatments, cells carrying circuit plasmids were diluted 1:6 in PBS and sampled from 96-well plates using a high-throughput sampler.
Instrument	BD FACSCelesta
Software	Data was collected with BD FACSDiva 8.01. and analysed with FCS Express 7
Cell population abundance	No sorting was performed.

Gating strategy	Electronic noise and cell debris were excluded gating FSC-A vs SSC-A, cell aggregates were excluded using FSC-A vs FSC-H and RFP positive cells were further gated by plotting RFP vs FSC-A

☒ Tick this box to confirm that a figure exemplifying the gating strategy is provided in the Supplementary Information.
