# Supplementary figures and images for: Interpretation of morphogen gradients by a synthetic bistable circuit
Source: Nat Commun. 2020 Nov 2;11:5545. doi: 10.1038/s41467-020-19098-w (PMC7608687; doi:10.1038/s41467-020-19098-w)

191119-C12

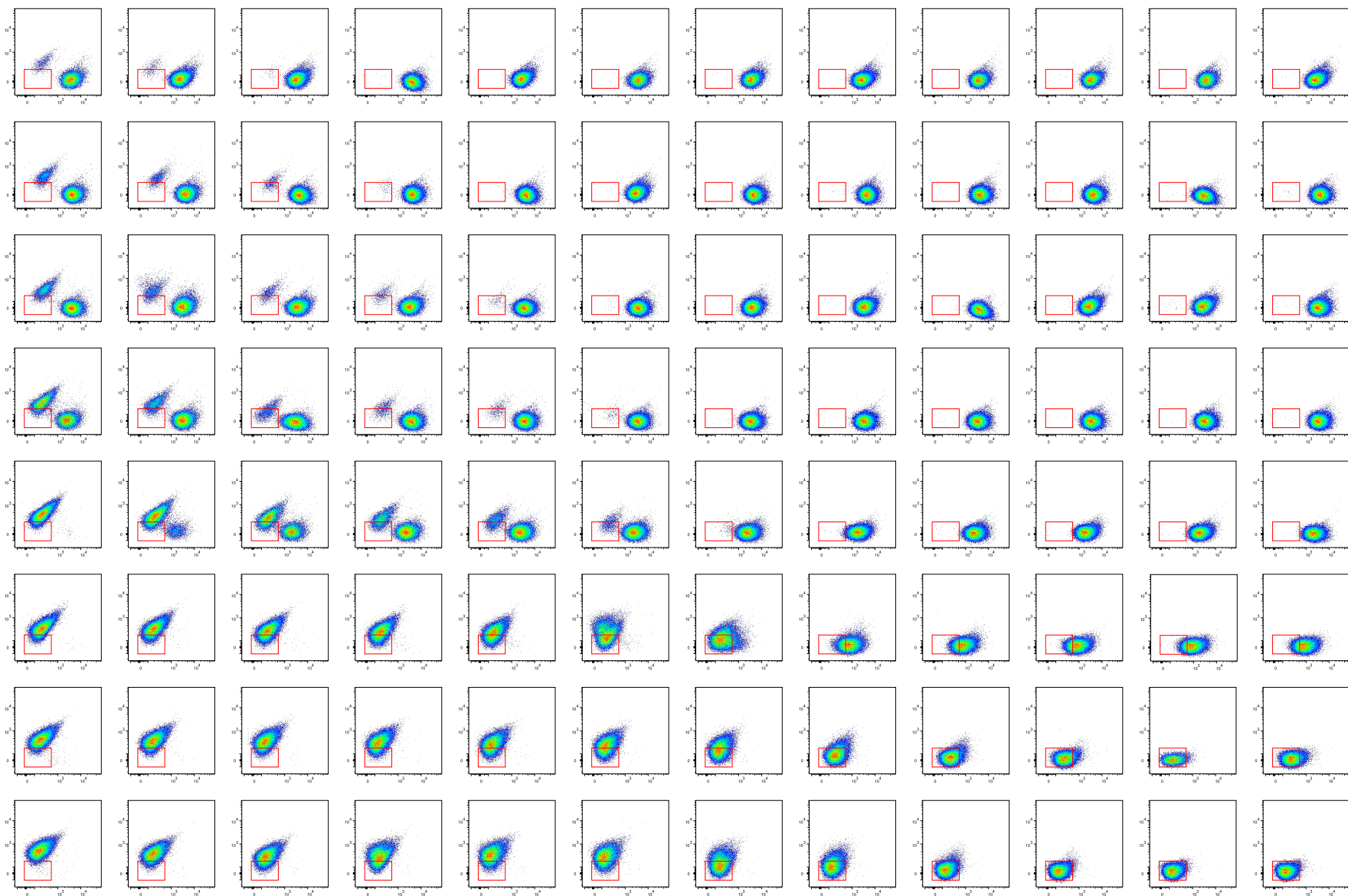

Supplement: Supplementary file 4 — Source Data [file 41467_2020_19098_MOESM4_ESM.zip › Source Data/Figs 2a S5 S13 FCS files/C12 Conditioned A/191119-Layout-C12.pdf]

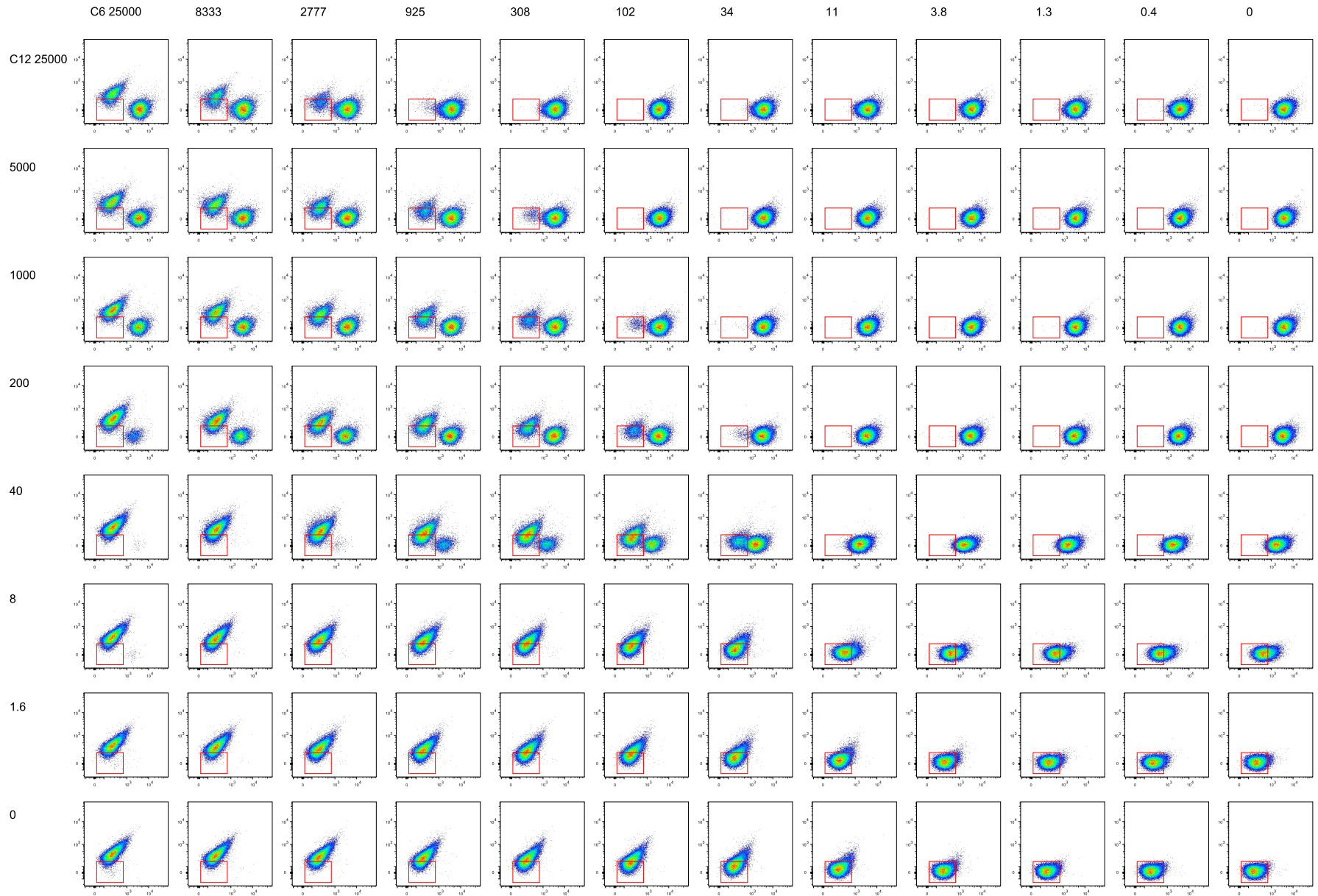

Supplement: Supplementary file 4 — Source Data [file 41467_2020_19098_MOESM4_ESM.zip › Source Data/Figs 2a S5 S13 FCS files/C12 Conditioned B/121219-Layout-C12.pdf]

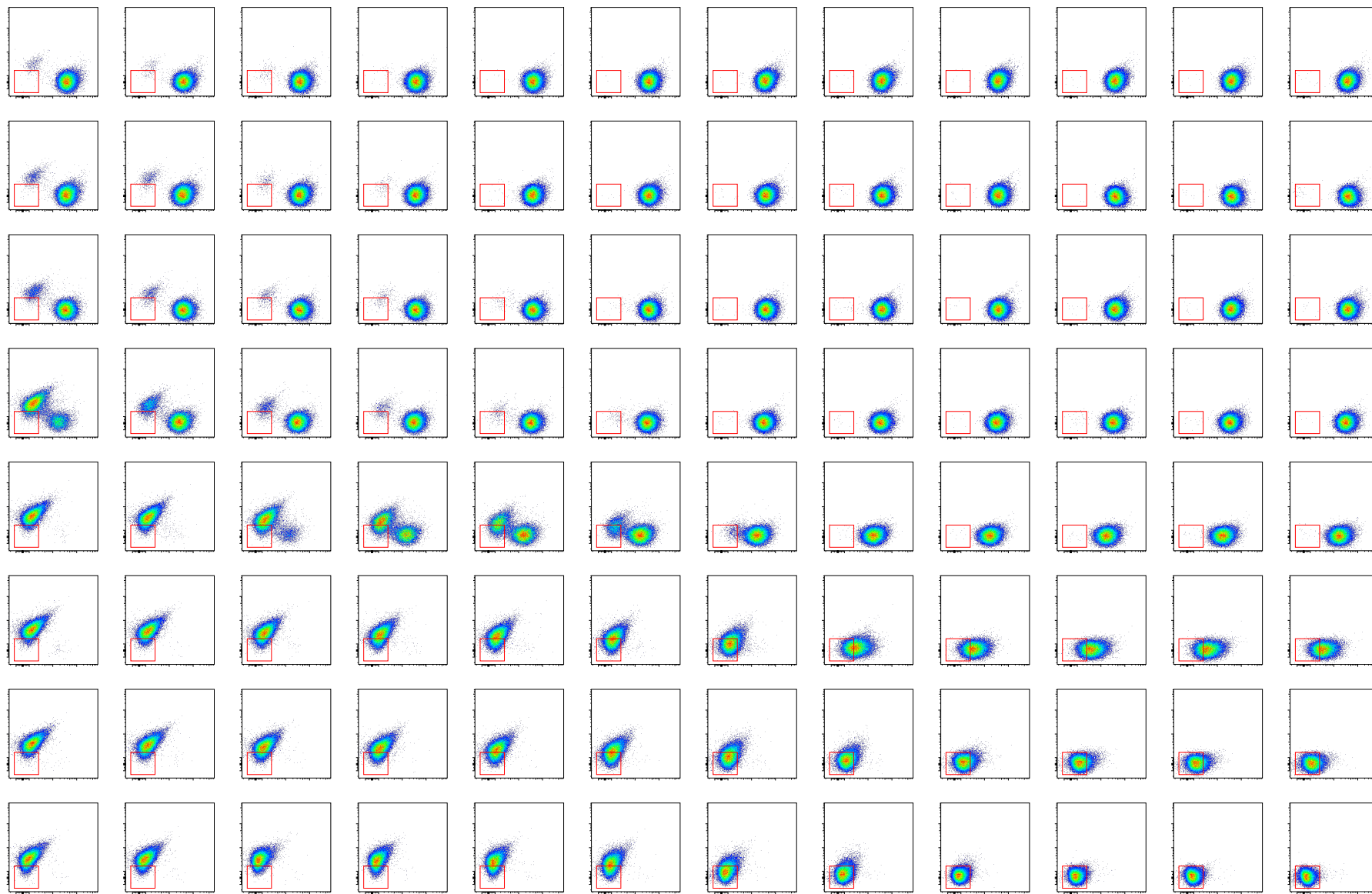

Supplement: Supplementary file 4 — Source Data [file 41467_2020_19098_MOESM4_ESM.zip › Source Data/Figs 2a S5 S13 FCS files/C12 Conditioned C/060220-Layout-C12.pdf]

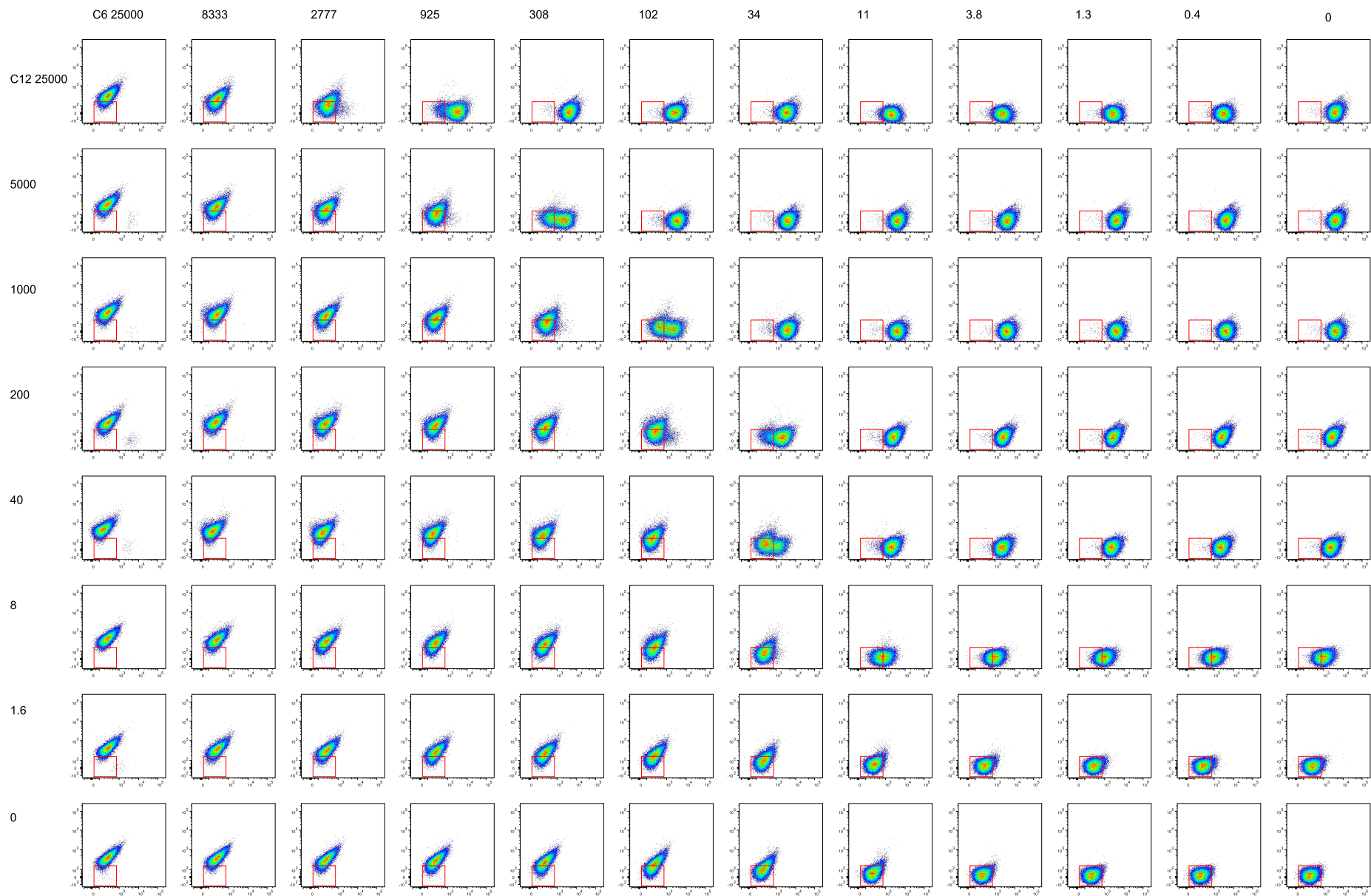

Supplement: Supplementary file 4 — Source Data [file 41467_2020_19098_MOESM4_ESM.zip › Source Data/Figs 2a S5 S13 FCS files/C6 Conditioned A/251119-Layout-C6.pdf]

171219-C6

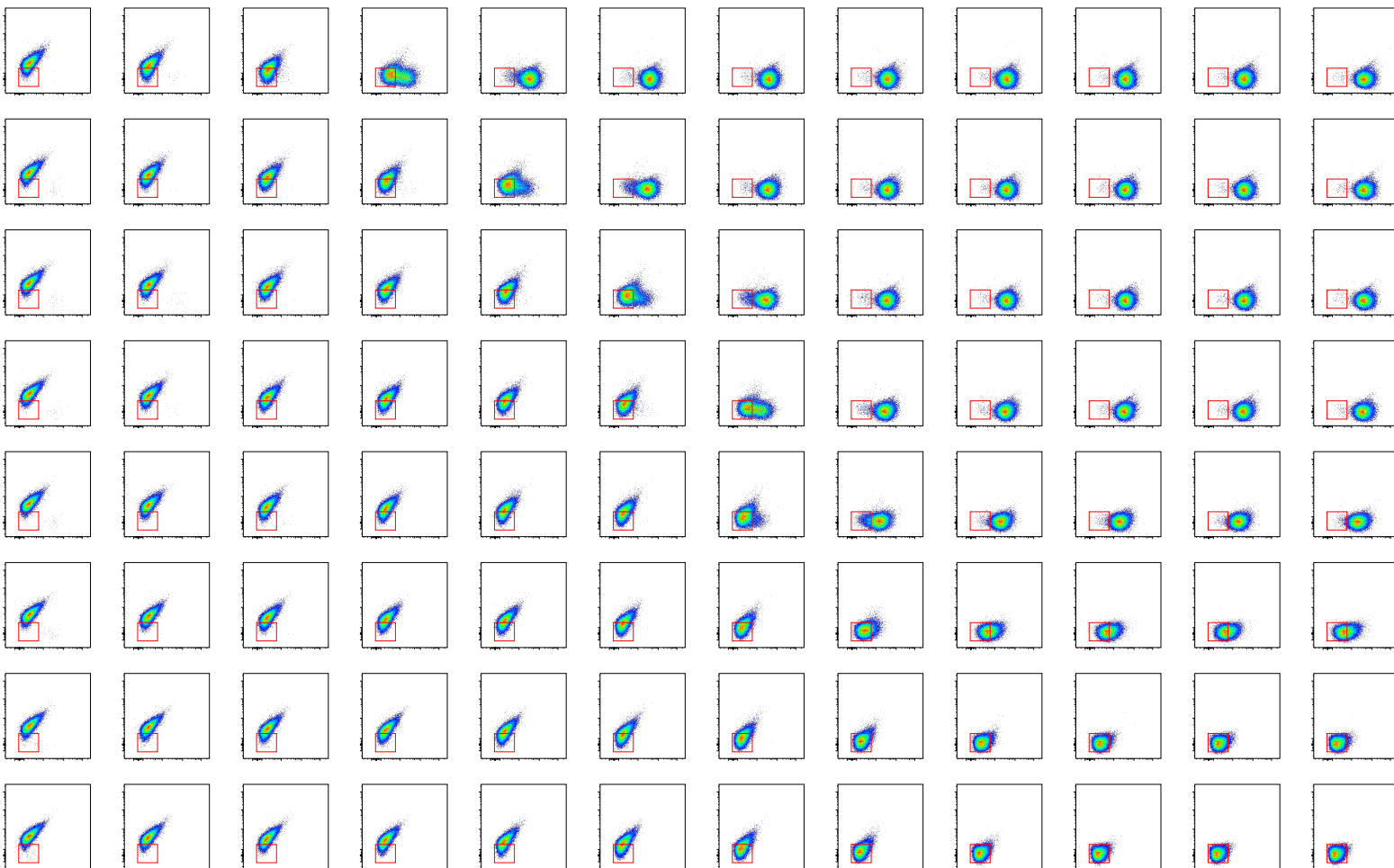

Supplement: Supplementary file 4 — Source Data [file 41467_2020_19098_MOESM4_ESM.zip › Source Data/Figs 2a S5 S13 FCS files/C6 Conditioned B/171219-Layout-C6.pdf]

080220 C6 primed

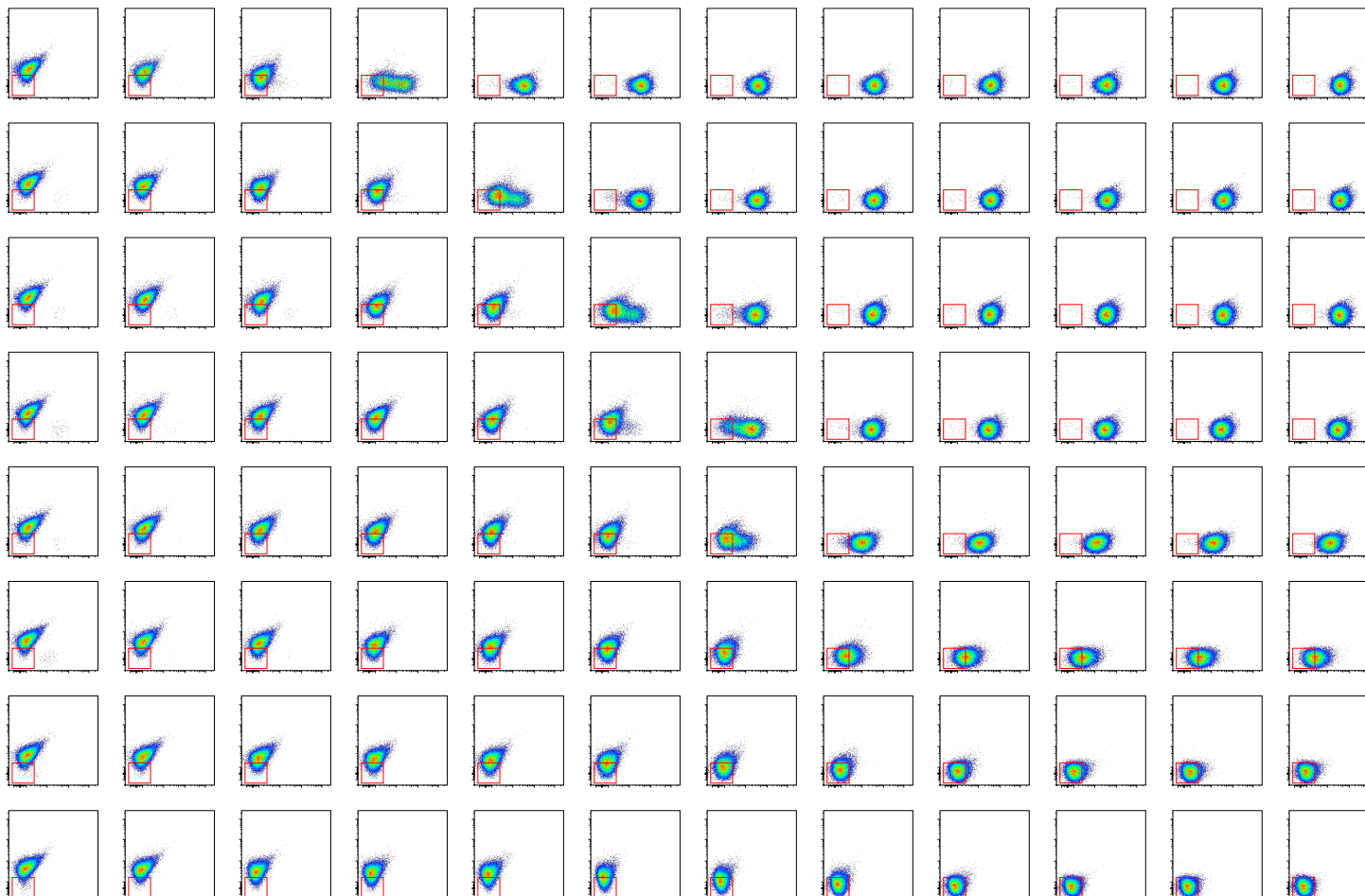

Supplement: Supplementary file 4 — Source Data [file 41467_2020_19098_MOESM4_ESM.zip › Source Data/Figs 2a S5 S13 FCS files/C6 Conditioned C/080220-C6.pdf]
